# Phytochemical Variability of *Mentha* L. Species Over Three Growing Seasons

**DOI:** 10.1002/cbdv.202503674

**Published:** 2026-03-02

**Authors:** Helena Pluháčková, Barbora Kudláčková, Marián Šinko, Markéta Michutová, Jarmila Neugebauerová, Lenka Svojanovská

**Affiliations:** ^1^ Department of Crop Science, Breeding and Plant Protection Mendel University in Brno Brno Czech Republic; ^2^ Institute of Analytical Chemistry of the Czech Academy of Sciences Brno Czech Republic; ^3^ Landscape Research Institute Pruhonice Czech Republic; ^4^ Department of Vegetable Growing and Floriculture Mendel University in Brno Brno Czech Republic

**Keywords:** gas chromatography, liquid chromatography, *Mentha* L. species, phytochemistry, UV/Vis spectroscopy

## Abstract

Species of the genus *Mentha* are valued for their aroma and bioactivity. This study presents a comprehensive 3‐year (2022–2024) evaluation of two *Mentha spicata* and three *Mentha × piperita* genotypes. Methanolic extracts were analyzed for total phenolic content (TPC), total flavonoid content (TFC), and antioxidant activity (AA) using spectrophotometry; individual phenolic compounds were determined by HPLC–DAD, and essential oils (EOs) were characterized by GC–MS. Significant inter‐ and intra‐species variability was observed*. M. × piperita* showed higher phytochemical levels (TPC: 33.61–55.23 mg GAE/g DW; TFC: 27.56–43.01 mg CE/g DW; and AA: 46.48–103.93 mg TE/g DW) than *M. spicata* (TPC: 28.92–39.57 mg GAE/g DW; TFC: 23.48–35.11 mg CE/g DW; and AA: 40.09–93.59 mg TE/g DW). Rosmarinic acid predominated in all samples (6227.11 to 29321.70 µg/g DW), with elevated levels observed in *M. × piperita*. EO yields ranged from 0.55% to 2.31% (v/w), with carvone dominant in *M. spicata* and menthone, dl‐menthol, or piperitenone oxide in *M. × piperita*. Principal component analysis confirmed distinct chemical differentiation among genotypes. Genotype had a strong, and year a moderate, effect on phytochemical profiles. These results highlight the species‐specific potential of *Mentha* for food, pharmaceutical, and cosmetic applications.

## Graphical abstract

Species of the genus *Mentha* are evaluated over three years for phytochemical composition and bioactivity using spectrophotometric, HPLC‐DAD, and GC‐MS analyses of methanolic extracts and essential oils. Clear genotype‐dependent differences are observed, with *Mentha* × *piperita* showing higher phenolic content and antioxidant activity than *M*. *spicata*. Genotype has a strong influence, while year has a moderate influence, highlighting the potential of specific species for use in food, pharmaceuticals, and cosmetics.

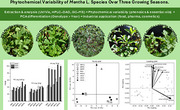

## 1 Introduction

Herbs and spices have played a vital role in human life since prehistoric times, especially due to their use in traditional medicine. The genus *Mentha* (Lamiaceae family), commonly known as mint, is widely distributed in Europe, Asia, Africa, Australia, and North America [1]. The taxonomy of the genus *Mentha* is complex, comprising approximately 42 species and 15 hybrids, along with numerous subspecies and cultivars [1, 2, 3]. This complexity is largely due to frequent intra‐ and inter‐specific hybridization and vegetative propagation. Generally, it has been suggested that the five basic species comprising the genus *Mentha* (*M. arvensis* L., *M. aquatica* L., *M. spicata* L., *M. longifolia* L., and *M. suaveolens*) have given rise to 11 naturally occurring and named hybrids [1, 2, 4].

A wide range of *Mentha* L. species are frequently utilized due to their diverse biological activities, including antioxidant, antimicrobial, anticancer, antiviral, antiallergic, anti‐inflammatory, antihypertensive, and biopesticidal effects [1, 2, 3, 4, 5]. These properties support their broad application across various sectors, such as pharmaceuticals, cosmetics, food, flavoring, beverages, and agriculture [1, 2, 3, 4, 5]. Among *Mentha* L. species, only four are reported to be cultivated commercially for their essential oils (EOs) and herbage yields: *M. spicata* L., *Mentha* × *piperita* L., *M. citrata* L. Erh, and *M. arvensis* L. [4]. The world's top producers, exporters, and consumers of *Mentha* oil are India (fulfills approximately 80% of international demand), together with the United States and China [6]. The biological activities associated with mint are attributed to its content of bioactive phytochemicals, which include mainly terpenoids, phenolic acids, and flavonoids. These metabolites, derived from the plant secondary pathways, show very high chemical diversity across *Mentha* chemotypes and ecotypes [2, 7, 8].

The chemical composition of *Mentha* can vary depending on many factors, such as growth location, soil properties, temperature, life cycle stage, plant variety, growth, climate conditions, and harvest time. Despite this variability, each *Mentha* species is generally defined by a characteristic main compound [7].

*M. spicata*, commonly known as spearmint, can be used fresh, dried, powdered, or traditionally as an herbal tea. In folk medicine, it is frequently used to treat respiratory and digestive issues. In addition to its use in pharmaceutical preparations, it is of great importance in the perfumery, cosmetics, and food industry [5]. The volatile profile of traditional cultivars of spearmint EOs is mainly constituted by carvone, along with other compounds, such as limonene, pulegone, piperitone, and other minor compounds. On the contrary, polar extracts of spearmint are characterized by a high content of phenolic compounds, such as rosmarinic acid, caffeic acid, *p*‐coumaric acid, apigenin, luteolin, and myricetin [2, 5, 8].

*M. *×* piperita*, a first‐generation hybrid between *M. aquatica* and *M. spicata*, is commonly known as peppermint and represents one of the most widely consumed single ingredient of herbal teas. Its beneficial actions include antioxidant and antimicrobial properties and benefits to the digestive tract. As one of the greatest possible sources of biologically active compounds for the food, cosmetics, and pharmaceutical industries, it is highly significant from an economic perspective [3]. Its EO is one of the most produced and marketed EOs worldwide. It is estimated that over 50% of EO produced are used for chewing gum, and about 30% are utilized in toothpaste and other oral care products. The distribution of menthol and menthone, as the major components of peppermint EO, may vary over a wide range from 30% to 60% and 10% to 30%, respectively. Rosmarinic acid, caffeic acid, gallic acid, rutin, hesperidin, and luteolin 7‐*O*‐glucuronide have been reported as major phenolic elements of *M. piperita* extract [2, 3, 8].

Despite the extensive use of mints, systematic multi‐year comparisons of their phytochemical profiles are still lacking. Most previous studies have focused on single seasons or specific chemotypes, providing only a limited perspective on phytochemical variability. However, the accumulation of secondary metabolites is influenced by genotype as well as environmental conditions, including year‐to‐year fluctuations in temperature and precipitation, which may lead to variation in phytochemical content due to changes in biosynthetic activity. A 3‐year study therefore offers a more robust basis for evaluating both species‐specific patterns and the impact of seasonal variation. On the basis of these, the present study aimed to characterize two species of *Mentha* L. cultivated in the Czech Republic during 2022–2024. The extracts were analyzed to compare their chemical composition using UV/Vis spectrophotometry for the determination of total phenolic content (TPC), total flavonoid content (TFC), and antioxidant activity (AA) by the DPPH radical assay. Individual phenolic compounds were identified and quantified by HPLC–DAD, whereas the EO profiles of the *Mentha* L. species were determined using GC–MS. Multivariate relationships among samples were assessed via principal component analysis (PCA).

## 2 Results and Discussion

### 2.1 UV/Vis Measurements

**FIGURE 1:**
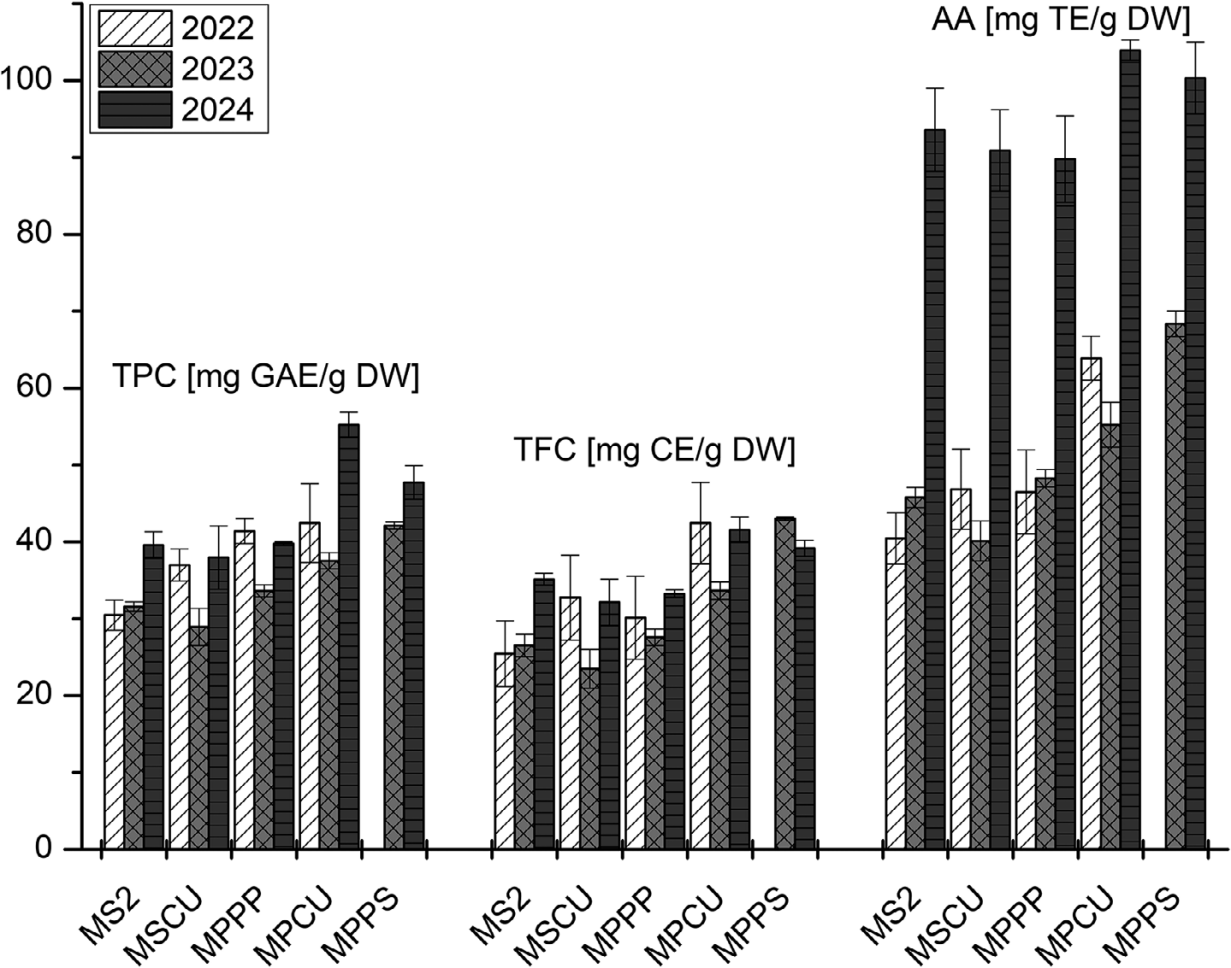
Results of UV/Vis measurements (TPC, TFC, AA) for all *Mentha* samples during 2022, 2023, and 2024. TPC = total phenolic content in mg gallic acid equivalent/g of dry weight, TFC = total flavonoids content in mg catechin equivalent/g of dry weight, AA = antioxidant activity in mg Trolox equivalent/g of dry weight. Results are expressed as mean value with error bars (*n* = 3). MPCU, *Mentha × piperita* “Corvinus University”; MPPP, *Mentha × piperita* “Perpeta”; MPPS, *Mentha × piperita* “Persephone”; MS2, *Mentha spicata* “2”; MSCU, *Mentha spicata* “Corvinus University.”

The obtained results of TPC, TFC, and AA of the methanolic mint extracts collected over three consecutive years are summarized in Figure 1 and Table S2. In 2022, *Mentha spicata* “2” (MS2) recorded the lowest levels of all analyzed parameters (30.47 ± 0.96 mg GAE/g DW for TPC, 25.44 ± 2.24 mg CE/g DW for TFC, and 40.46 ± 2.30 mg TE/g DW for AA), whereas *M. × piperita* “Corvinus University” (MPCU) showed the highest values (42.43 ± 2.15 mg GAE/g DW, 42.44 ± 2.30 mg CE/g DW, and 63.90 ± 2.87 mg TE/g DW). A different pattern emerged in 2023, when *Mentha spicata* “Corvinus University” (MSCU) exhibited the lowest TPC, TFC, and AA values (28.92 ± 2.42 mg GAE/g DW, 23.48 ± 2.50 mg CE/g DW, and 40.09 ± 2.51 mg TE/g DW), whereas *M. × piperita* “Persephone” (MPPS) reached the highest levels across all parameters (42.12 ± 0.43 mg GAE/g DW, 43.01 ± 0.24 mg CE/g DW, and 68.36 ± 1.68 mg TE/g DW). In 2024, all species showed a clear increase in the measured parameters, particularly in AA. In that year, MSCU again displayed the lowest TPC and TFC values (37.96 ± 2.10 mg GAE/g DW and 32.13 ± 2.83 mg CE/g DW), whereas MPCU exhibited the highest TPC, TFC, and AA values (55.23 ± 1.65 mg GAE/g DW, 41.57 ± 1.68 mg CE/g DW, and 103.93 ± 1.33 mg TE/g DW). These results demonstrate clear genotypic differences and year‐dependent effects on phenolic accumulation and antioxidant potential.

Similar results were previously reported by Bouali et al. [9] in *M. spicata* ethyl acetate (EtAc) and chloroformic extracts (Chl), where TPC was found at 65.06 ± 1.10 (EtAc) and 52.1 ± 1.22 mg GAE/g DW (Chl) and TFC at 40.83 ± 1.25 (EtAc) and 32.6 ± 1.10 mg CE/g DW (Chl). Bellik and Selles [10] found average TPC at 25.49 ± 0.07 mg GAE/g and TFC at 29.76 ± 0.39 mg QE/g in *M. spicata* water extract. The TPC, TFC, and AA values of methanolic mint extracts published by Cavar Zeljkovic et al. [11], who found TPC from 14.81 ± 1.09 to 58.93 ± 8.39 mg GAE/g; TFC from 3.65 ± 0.037 to 16.83 ± 1.45 mg QE/g and AA from 22.79 ± 1.85 to 106.04 ± 3.26 mg TE/g are in good agreement with presented data. The TPC, TFC, and AA values of the alcoholic extracts of investigated *Mentha* species had already been studied elsewhere, but the values differed significantly [11]. This can be attributed to differences in extraction solvents, analytical protocols, and various biotic and abiotic factors such as genotype, geographic origin, soil type, and cultivation conditions [1, 7, 11].

Our results align with this pattern and further suggest that interannual climatic variability played an important role in the observed year‐to‐year differences. In particular, the exceptionally high TPC, TFC, and AA values in 2024 correspond with a season characterized by higher mean temperatures and increased precipitation (Figure S1), which are known to stimulate phenolic biosynthesis in Lamiaceae. These results suggest that secondary metabolite accumulation in *Mentha* is not solely genotype‐driven but is also partially modulated by prevailing environmental conditions during the growing season [12, 13, 14].

### 2.2 HPLC–DAD Analysis

A total of 13 phenolic compounds were determined by HPLC–DAD, including five flavonoids (rutin, myricetin, luteolin, quercetin, and apigenin) and eight phenolic compounds (protocatechuic acid, chlorogenic acid, *p*‐hydroxybenzoic acid, caffeic acid, syringic acid, ferulic acid, rosmarinic acid, and cinnamic acid). Significant variability in the content of phenolic compounds was observed both among the mint samples and across the three consecutive years of cultivation. Rosmarinic acid was the predominant compound in all samples, with concentrations ranging from 6227.11 ± 205.04 µg/g DW in MS2 (2022) to 29 320.70 ± 1026.75 µg/g DW in MPCU (2023) (Table 1). Other major constituents included chlorogenic acid, ferulic acid, and rutin, although their levels varied considerably depending on genotype and year. Marked interspecific differences were observed in the flavonoid profile. In particular, luteolin and quercetin were consistently detected in *M. spicata* at average concentration of 12.35 ± 0.53 µg/g DW, whereas these compounds were absent in *M. × piperita*, with the exception of MPPS in 2023, where luteolin was detected at 2.66 ± 0.13 µg/g DW (Table 1). The representative HPLC–DAD chromatograms are presented in Figures S2–S6.

**TABLE 1:** Content (µg/g) of phenolic compounds in *Mentha* extracts identified and quantified by HPLC‐DAD.

Compound	MS2	MS2	MS2	MSCU	MSCU	MSCU	MPPP	MPPP	MPPP	MPCU	MPCU	MPCU	MPPS	MPPS
	2022	2023	2024	2022	2023	2024	2022	2023	2024	2022	2023	2024	2023	2024
Protocatechuic acid	8.46^b^	8.95^b^	—	—	8.68^b^	—	—	—	—	9.05^b^	9.23^b^	10.85^c^	—	—
	± 0.02	± 0.21			± 0.01					± 0.10	± 0.10	± 0.64		
Chlorogenic acid	237.27^a^	500.24^d^	918.51^g^	305.99^b^	612.82^e^	920.39^g^	282.58^ab^	470.39^cd^	423.56^c^	821.62^f^	971.03^g^	938.92^g^	964.82^g^	670.74^e^
	± 12.38	± 8.87	± 11.64	± 2.34	± 14.17	± 0.10	± 5.35	± 5.41	± 11.33	± 3.62	± 28.44	± 0.19	± 9.42	± 3.84
*p*‐Hydroxybenzoic acid	—	9.99^b^	—	11.04^b^	9.66^b^	—	—	—	—	16.46^c^	22.48^e^	19.78	14.31^c^	—
		± 0.20		± 0.59	± 0.10					± 1.23	± 0.42	± 0.31	± 0.32	
Caffeic acid	81.26^a^	103.99^cdef^	111.17^efgh^	93.04^abc^	107.57^defgh^	119.52^gh^	96.04^bcd^	97.59^bcde^	109.16^defgh^	106.30^cdefg^	117.95^fgh^	191.30	120.99^h^	85.50^ab^
	± 1.30	± 1.09	± 0.26	± 2.93	± 0.10	± 1.76	± 2.67	± 1.52	± 1.32	± 3.64	± 2.46	± 1.00	± 6.38	± 1.29
Syringic acid	45.65^b^	69.87^d^	61.44^c^	62.19^cd^	64.15^cd^	62.75^cd^	—	—	—	—	—	—	68.72^cd^	63.45^cd^
	± 0.78	± 0.99	± 2.27	± 2.41	± 1.41	± 1.48							± 2.92	± 2.04
Rutin	710.93^d^	920.34^ef^	1010.32^f^	859.90^e^	886.05^ef^	1419.24^g^	365.31^a^	502.98^b^	303.63^a^	576.56^bc^	669.25^cd^	624.43	267.02^a^	289.67^a^
	± 27.71	± 30.21	± 16.10	± 52.10	± 35.92	± 22.91	± 3.73	± 7.30	± 4.12	± 14.56	± 9.86	± 0.07	± 10.76	± 2.72
Ferulic acid	2497.93^c^	3627.55	3280.21^ef^	3269.21^ef^	3088.26^de^	5463.40^g^	1708.86^b^	1753.74^b^	2365.34^c^	—	—	—	3100.42^de^	2750.66^cd^
	± 110.85	± 12.69	± 172.77	± 202.69	± 87.11	± 2.30	± 19.00	± 0.97	± 82.08				± 29.89	± 22.30
Rosmarinic acid	6227.11^a^	10265.37^bcd^	8379.91^ab^	8424.68^abc^	10049.66^bcd^	11363.00^cde^	13279.69^ef^	14608.76^fg^	12478.04^def^	17804.46^h^	29320.70^j^	27543.53	22270.83^i^	17545.01^gh^
	± 205.04	± 287.15	± 237.70	± 398.11	± 340.98	± 172.22	± 1464.31	± 136.94	± 252.81	± 84.68	± 1026.75	± 58.79	± 75.69	± 205.68
Myricetin	—	—	—	—	—	—	—	—	—	79.21^c^	75.02^bc^	69.40	415.92^e^	229.85^d^
										± 0.23	± 0.17	± 0.89	± 4.62	± 1.00
Luteolin	8.44^b^	12.00^cd^	15.64^e^	14.67^de^	11.00^bc^	18.53^f^	—	—	—	—	—	—	2.66^a^	—
	± 0.31	± 0.23	± 0.41	± 1.61	± 0.51	± 0.34							± 0.13	
Quercetin	11.67^bc^	11.52	10.81^bc^	11.31^bc^	9.76^b^	12.85^c^	—	—	—	—	—	—	—	—
	± 0.28	± 1.17	± 0.82	± 0.37	± 0.01	± 0.28								
Cinnamic acid	—	—	—	—	—	—	2.89^b^	4.44^c^	2.89^b^	—	—	—	—	—
							± 0.10	± 0.07	± 0.06					
Apigenin	31.62^g^	27.99	13.19^b^	39.88^h^	32.15^g^	16.21^bc^	18.16^cd^	16.31^bc^	4.96^a^	21.61^de^	25.14^ef^	15.63	26.55^f^	13.84^bc^
	± 2.04	± 0.51	± 0.21	± 1.25	± 0.09	± 0.16	± 1.02	± 0.25	± 0.09	± 1.07	± 1.08	± 0.48	± 0.72	± 0.16

Values (µg/g) are expressed as mean ± standard deviation (n = 3). MS2 ‐ *Mentha spicata* ‘2’; MSCU ‐ *Mentha spicata* ‘Corvinus University’; MPPP – *Mentha × piperita* ‘Perpeta’; MPCU – *Mentha × piperita* ‘Corvinus University’. Means followed by the same letter are not statistically different at p ≤ 0.05 according to Tukey's HSD test.

Part of the observed interannual variability in phenolic content can be attributed to differences in climatic conditions during the three growing seasons. The elevated concentrations of rosmarinic and chlorogenic acids recorded in 2024 correspond with warmer temperatures and higher precipitation levels documented for that year (Figure S1), conditions known to stimulate flux through the phenylpropanoid pathway. Although genotype remained the primary determinant of phenolic composition, environmental factors appear to have modulated the absolute concentrations of individual compounds across seasons.

The obtained results are in good agreement with those reported by Farahbakhsh et al. [15], who found 19.64 mg/g of rosmarinic acid in a methanolic extract of *M. spicata*, as well as by Kosar et al. [16], who reported rosmarinic acid levels ranging from 0.66 ± 0.02 to 12.11 ± 0.12 mg/g in water‐soluble *Mentha* extracts. According to recent reviews, phenolic acids, such as rosmarinic, caffeic, and ferulic acid, are one of the most important active compounds in *Mentha* species [1, 2, 5].

Consistent with previous findings, although substantial quantitative differences were observed among samples, rosmarinic acid remained the dominant phenolic compound across all genotypes and years.

### 2.3 EO Yields and GC–MS Characterization of EOs

The average EO yields from three independent hydrodistillations of each sample are presented in Table 2. The EO yields ranged from 0.55% to 2.31% (v/w), with the highest yields observed in 2024 for all species. These results are in good agreement with previously reported data [3, 17, 18]. Hosseini et al. [18] applied salinity stress (three NaCl applications) to increase the EO content. *M. × piperita* showed the highest content of EOs (2.94%, v/w) compared to the control (0.61%, v/w).

**TABLE 2:** Essential oil (EO) yields for all *Mentha* samples during 2022–2024.

Yield (%)	MS2	MSCU	MPPP	MPCU	MPPS
**2022**	0.67^c^ ± 0.00	0.83^c^ ± 0.02	0.57^a^ ± 0.02	2.02^d^ ± 0.02	—
**2023**	1.29^c^ ± 0.00	0.96^b^ ± 0.04	1.16^c^ ± 0.02	1.76^d^ ± 0.02	0.55^a^ ± 0.04
**2024**	1.90^c^ ± 0.03	1.88^c^ ± 0.07	1.23^b^ ± 0.09	2.31^d^ ± 0.05	0.77^a^ ± 0.03

*Note*: Results are expressed as mean value ± standard deviation (*n* = 3). Yield (%, v/w). Means followed by the same letter are not statistically different at *p* ≤ 0.05 according to Tukey's HSD test.

Abbreviations: MPCU, *Mentha × piperita* “Corvinus University”; MPPP, *Mentha × piperita* “Perpeta”; MPPS, *Mentha × piperita* “Persephone”; MS2, *Mentha spicata* “2”; MSCU, *Mentha spicata* “Corvinus University.”

The EO composition, like the other measurements, also showed notable differences. A total of 67 components were identified in the *Mentha* EOs. For better clarity, compounds present at levels above 1% are displayed in Table 3, and full dataset is provided in Tables S3–S5. The GC–MS chromatograms of the representative samples are shown in Figure S7. The abundance of most substances varied not only among species but also between species of *M. × piperita*. The main component in each species was identified as follows: MS2‐carvone (±68%), MSCU‐carvone (±65%), *M. × piperita* “Perpeta” (MPPP)‐menthone (±58%), MPCU‐dl‐menthol (±50%), and MPPS‐piperitenone oxide (±67%) (Table 3). The difference in the content of the main compound over the years was not significant for all species. However, there are differences in the content of other compounds during the years; for example, the MPPS species, where the content of the germacrene D during 2023–2024 was as follows: 14.80% ± 0.82%, 5.68% ± 3.33%, and 60.13% ± 2.75%, respectively. This suggests that certain species are more sensitive to environmental fluctuations, consistent with previous reports linking EO composition to climate and soil conditions [1, 2, 5]. Chrysargyris et al. [12] demonstrated that different potassium level applications during the *M. spicata* growth influenced plant growth, mineral content, antioxidant and antibacterial activities, and changes in the constituents of EOs. Karami et al. [19] confirmed the influence of drying temperature (40°C–60°C) on the EO content of *Mentha* and recommended maintaining a temperature of 40°C. Smitha and Rana [20] stated that the viral disease and the presence of whiteflies have significantly reduced the amount of biomass and EO content in *Mentha*, but they have had less of an impact on the levels of menthol, menthone, limonene, and carvone. Pérez‐Vázquez et al. [21] revealed that the levels of principal volatiles (menthone and menthol) of *M. × piperita* EO remained relatively constant during the seasonal variation (four seasons in 2020), but high variation in minor volatiles (e.g., 1,4‐cineole, isomenthol, and spathulenol) was observed.

**TABLE 3:** Chemical composition of essential oils (% ± SD) in all *Mentha* samples during 2022–2024 determined by GC‐MS.

PeakNo.	RI Ca	RI Ad	Compound^1)^	MS2	MS2	MS2	MSCU	MSCU	MSCU	MPPP	MPPP	MPPP	MPCU	MPCU	MPCU	MPPS	MPPS
				2022	2023	2024	2022	2023	2024	2022	2023	2024	2022	2023	2024	2023	2024
3	982	974	*β*‐Pinene	0.11^a^	0.23^abc^	0.40^bc^	0.16^a^	0.19^ab^	0.44^c^	0.29^abc^	1.14^d^	0.96^d^	0.16^a^	0.14^a^	0.31^abc^	0.10^a^	0.21^abc^
				± 0.01	± 0.00	± 0.01	± 0.02	± 0.02	± 0.04	± 0.06	± 0.02	± 0.11	± 0.01	± 0.00	± 0.03	± 0.01	± 0.08
8	1033	1024	Limonene	0.83^abc^	4.87^g^	6.72^h^	1.44^de^	2.08^f^	7.05^h^	0.62^ab^	0.96^bcd^	0.92^bc^	0.72^ab^	1.54^e^	1.25^cde^	0.34^a^	0.37^a^
				± 0.01	± 0.02	± 0.08	± 0.03	± 0.22	± 0.13	± 0.01	± 0.03	± 0.03	± 0.01	± 0.01	± 0.07	± 0.00	± 0.15
9	1037	1026	Eucalyptol^2)^	0.31^ab^	0.70^abc^	1.14^bcd^	0.37^abc^	0.44^abc^	0.74^abc^	1.72^d^	3.64^e^	3.50^e^	0.04^a^	0.05^a^	0.36^ab^	1.88^d^	1.30^cd^
				± 0.02	± 0.05	± 0.01	± 0.04	± 0.01	± 0.09	± 0.03	± 0.02	± 0.11	± 0.02	± 0.01	± 0.03	± 0.02	± 0.20
11	1075	1065	*cis*‐Sabinene hydrate	0.16^abc^	0.35^c^	0.10^ab^	0.15^abc^	0.21^abc^	0.13^abc^	0.86^d^	1.04^d^	0.28^bc^	0.04^a^	0.03^a^	—	0.17^abc^	0.02^a^
				± 0.02	± 0.06	± 0.02	± 0.00	± 0.01	± 0.01	± 0.05	± 0.14	± 0.05	± 0.01	± 0.01		± 0.00	± 0.01
15	1163	1148	Menthone	3.35^a^	0.51^a^	0.27^a^	0.10^a^	1.57^a^	0.31^a^	53.76^d^	63.57^e^	57.21^d^	23.82^b^	29.70^c^	32.29^c^	—	1.81^a^
				± 0.09	± 0.18	± 0.03	± 0.00	± 0.39	± 0.03	± 1.98	± 0.77	± 1.84	± 0.67	± 0.42	± 0.59		± 0.68
16	1171	1159	Menthofuran	0.03^a^	0.03^a^	0.02^a^	0.01^a^	0.02^a^	0.03^a^	1.21^b^	1.26^b^	1.25^b^	—	—	—	—	0.49
				± 0.01	± 0.01	± 0.01	± 0.00	± 0.01	± 0.01	± 0.25	± 0.24	± 0.15					± 0.17
17	1172	1158	Iso‐menthone	0.34^a^	0.13^a^	0.03^a^	0.03^a^	0.39^a^	0.02^a^	3.94^b^	5.70^c^	4.52^b^	7.34^d^	9.88^e^	10.14^e^	—	—
				± 0.01	± 0.01	± 0.01	± 0.01	± 0.12	± 0.01	± 0.06	± 0.12	± 0.30	± 0.36	± 0.07	± 0.00		
18	1176	1161	Neo‐menthol	0.79^bc^	0.23^abc^	0.11^ab^	—	0.25^abc^	—	19.41^f^	10.05^e^	8.72^d^	0.93^c^	0.65^abc^	0.36^abc^	—	0.40^abc^
				± 0.04	± 0.05	± 0.01		± 0.11		± 0.05	± 0.20	± 0.32	± 0.02	± 0.06	± 0.04		± 0.18
19	1183	1167	DL‐Menthol	—	0.24^a^	—	—	1.03^a^	—	0.29^a^	0.20^a^	0.19^a^	56.21^c^	46.48^b^	45.85^b^	—	—
					± 0.04			± 0.31		± 0.04	± 0.01	± 0.01	± 0.75	± 1.60	± 2.55		
20	1187	1174	Terpinen‐4‐ol	0.19^abc^	0.19^abc^	0.14^abc^	0.13^ab^	0.15^abc^	0.16^abc^	0.51^bcd^	0.55^cd^	1.02^e^	—	—	—	0.10^ab^	0.71^de^
				± 0.01	± 0.00	± 0.01	± 0.01	± 0.05	± 0.01	± 0.06	± 0.08	± 0.06				± 0.01	± 0.25
23	1204	1191	*cis*‐Dihydrocarvone	12.17^d^	7.28b^c^	7.32b^c^	12.08^d^	6.60^b^	10.20^cd^	0.53^a^	—	0.06^a^	—	—	—	0.09^a^	—
				± 1.45	± 0.02	± 0.07	± 0.21	± 0.93	± 0.00	± 0.07		± 0.01				± 0.02	
27	1246	1233	Pulegone	0.08^a^	—	—	—	—	—	2.09^b^	0.35^a^	4.55^c^	—	—	—	—	0.21^a^
				± 0.01						± 0.31	± 0.01	± 0.23					± 0.06
28	1252	1239	Carvone	68.00^bc^	71.37^c^	65.44^bc^	71.27^c^	64.82^bc^	60.13^b^	2.15^a^	—	2.43^a^	0.06^a^	0.68^a^	—	3.19^a^	0.20^a^
				± 0.70	± 0.23	± 1.78	± 0.68	± 3.30	± 2.75	± 0.47		± 0.14	± 0.02	± 0.20		± 0.89	± 0.02
29	1262	1249	Piperitone	0.09^ab^	0.23^ab^	—	—	0.19^ab^	—	2.17^c^	1.85^bc^	3.12^cd^	6.13^e^	6.44^e^	4.16^d^	0.08^ab^	0.22^ab^
				± 0.01	± 0.04			± 0.09		± 0.23	± 0.01	± 0.33	± 0.05	± 0.93	± 0.64	± 0.01	± 0.07
31	1276	1271	Neo‐menthyl acetate	0.08^a^	—	—	—	0.05	—	1.79^c^	1.09^b^	1.21^b^	—	0.08^a^	—	—	—
				± 0.01				± 0.03		± 0.10	± 0.10	± 0.09		± 0.02			
41	1369	1366	Piperitenone oxide	—	—	—	—	—	—	—	—	—	—	—	—	62.93^b^	70.48^c^
																± 3.16	± 2.74
44	1396	1387	*β*‐Bourbonene	1.00^bcd^	1.45^defg^	1.74^fgh^	2.38^h^	1.37^def^	2.14^gh^	—	0.42^ab^	1.05^bcde^	1.12^cdef^	1.13^cdef^	0.91^bcd^	1.70^efgh^	0.61^abc^
	+ 1398	+ 1389	+ *β*‐Elemene	± 0.19	± 0.04	± 0.01	± 0.02	± 0.31	± 0.22		± 0.05	± 0.11	± 0.06	± 0.01	± 0.03	± 0.01	± 0.08
45	1399	1392	*cis*‐Jasmone	0.90^cd^	0.49^b^	1.09^d^	0.99^d^	0.73^c^	0.91^cd^	—	—	—	—	—	—	0.41^b^	0.25^b^
				± 0.07	± 0.01	± 0.01	± 0.01	± 0.01	± 0.14							± 0.02	± 0.04
49	1434	1417	*β*‐Caryophyllene	2.31^bcd^	2.16^abcd^	1.76^abc^	2.47^bcd^	3.03^d^	2.81^cd^	2.39^bcd^	1.49^ab^	3.31^de^	1.32^ab^	1.03^a^	1.45^ab^	4.51^ef^	5.22^f^
				± 0.01	± 0.07	± 0.03	± 0.06	± 0.56	± 0.16	± 0.28	± 0.16	± 0.33	± 0.01	± 0.10	± 0.03	± 0.21	± 0.19
54	1475	1461	*cis*‐Cadina‐1(6),4‐diene	0.76^b^	0.73^b^	1.02^bc^	0.79	0.99^bc^	1.35^cd^	0.20^a^	0.13^a^	0.32^a^	—	—	—	1.70^d^	1.43^d^
				± 0.05	± 0.06	± 0.02	± 0.01	± 0.01	± 0.09	± 0.01	± 0.02	± 0.04				± 0.14	± 0.16
56	1494	1480	Germacrene D	1.52^ab^	1.84^ab^	2.33^abc^	1.49^ab^	4.36^abc^	2.01^abc^	2.93^abc^	2.01^abc^	4.80^bc^	0.67^a^	0.53^a^	0.83^a^	14.80^d^	5.68^c^
				± 0.09	± 0.02	± 0.03	± 0.14	± 1.34	± 0.14	± 0.27	± 0.20	± 0.30	± 0.01	± 0.05	± 0.05	± 0.82	± 0.33
60	1532	1528	*cis*‐Calamenene	0.88^bc^	0.74^b^	0.77^b^	0.89^bc^	1.19^c^	0.94^bc^	0.13^a^	0.05^a^	0.18^a^	—	—	—	0.90^bc^	0.93^bc^
				± 0.03	± 0.02	± 0.04	± 0.01	± 0.09	± 0.11	± 0.01	± 0.01	± 0.02				± 0.10	± 0.10
65	1630	1618	1,10‐di‐epi‐Cubenol	0.39^c^	0.38^c^	0.47^cd^	0.35^bc^	0.76^e^	0.63^de^	0.09^a^	0.07^a^	0.17^ab^	—	—	—	0.65^de^	1.01^f^
				± 0.04	± 0.01	± 0.02	± 0.02	± 0.08	± 0.00	± 0.00	± 0.02	± 0.02				± 0.05	± 0.09

Peak No. = Peak number, the numbering corresponds to Table S2‐S4; RI Ca = Retention index calculated with respect to homologous series of n‐alkanes (C9‐C23) on a DB‐5 column; RI Ad = Retention index from literature ‐ entered from Robert P. Adam's library. MS2 ‐ Mentha *spicata* ‘2’2; MSCU ‐ *Mentha spicata* ‘Corvinus University’; MPPP – *Mentha × piperita* ‘Perpeta’; MPCU – *Mentha × piperita* ‘Corvinus University’. Results are reported in % ± SD (standard deviation) as the mean of three experiments. Means followed by the same letter are not statistically different at p ≤ 0.05 according to Tukey's HSD test.

^1)^ Compounds that account less than 1% of EO are not presented.

^2)^ In sample MPPP, MPCU, and MPPS it is the sum of eucalyptol and ocimene (RI 1032).

In general, a number of factors affect *Mentha's* EO yield and oil composition, including the plant's genotype, origin, soil, climate, harvest stage, isolation techniques, farming conditions, and analytical procedures [1, 7, 11].

### 2.4 Statistical Analysis

**FIGURE 2:**
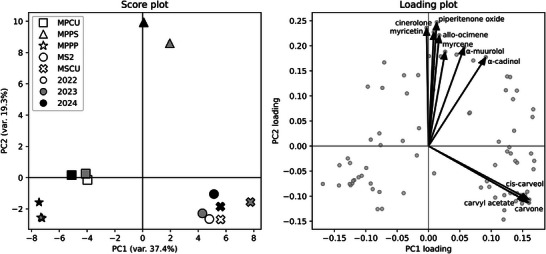
Principal component analysis (PCA) of phytochemical profiles of *Mentha spicata* and *Mentha × piperita* species evaluated over three growing seasons (2022–2024). The score plot (left) displays the samples projected onto the first two principal components, with PC1 explaining 37.4% and PC2 explaining 19.3% of the total variance. Species are coded by marker shape (square = MPCU‐*Mentha × piperita* “Corvinus University,” triangle = MPPS‐*Mentha × piperita* “Persephone,” star = MPPP‐*Mentha × piperita* “Perpeta,” circle = MS2‐*Mentha spicata* “2,” cross = MSCU‐*Mentha spicata* “Corvinus University”). Years are encoded by marker fill (2022‐white, 2023‐gray, 2024‐black). Loading plot (right) showing all variable loadings as gray points; the 10 variables with the highest absolute contribution to PC1/PC2 are highlighted by arrows and labeled.

PCA was applied to all phytochemical variables to evaluate multivariate relationships among *M. spicata* and *M. × piperita* species across three growing seasons (Figure 2). Prior to analysis, the data were autoscaled (mean‐centered and scaled to unit variance) to ensure that all variables, regardless of their absolute concentrations, contributed equally to the analysis. The score plot displays the group centroids for each species‐by‐year combination, providing an overview of multivariate similarity among sample groups. The first and second principal components together accounted for 56.7% of the total variance. The loading plot highlights the 10 variables contributing most strongly to sample separation, based on their absolute contribution to PC1/PC2. Loading plot of all variables by absolute contribution to PC1/PC2 is shown in Figure S8.

PCA revealed a clear separation between *M. spicata* and *M. × piperita* along PC1, which explained 37.4% of the total variance and primarily reflected differences in dominant monoterpenes and flavonoids. Separation along PC2 (19.3%) was observed mainly among *M. × piperita* species, with the MPPS forming a distinct cluster associated with variation in specific oxygenated monoterpenes. Samples from different years clustered consistently within genotype groups, indicating limited influence of interannual environmental variation.

The separation of *M. spicata* was driven mainly by higher levels of carvone and *cis‐*carveol, whereas *M. × piperita* was characterized by piperitenone oxide, myricetin, menthone, and dl‐menthol. Among *M. × piperita* species, the distinct positioning of MPPS along PC2 was driven predominantly by piperitenone oxide, indicating a unique secondary metabolite composition.

Overall, the consistent clustering of samples across three growing seasons demonstrates that species identities are the primary determinants of phytochemical composition, whereas interannual environmental variation plays a comparatively minor role, confirming the effectiveness of PCA in revealing biologically meaningful differences among *Mentha L*. species.

## 3 Conclusions

This study provided a multi‐year characterization of two *M. spicata* and three *M. × piperita* species using complementary spectrophotometric, chromatographic, and chemometric approaches. The results demonstrated clear inter‐ and intra‐specific differences in both phenolic composition and EO profiles. *M. × piperita* consistently accumulated higher levels of phenolics and exhibited stronger AA compared to *M. spicata*, with rosmarinic acid identified as the dominant compound in all species. EO yields and volatile profiles were strongly genotype‐dependent: Carvone was characteristic of *M. spicata*, whereas *M. × piperita* displayed distinct chemotypes dominated by piperitenone oxide, carvone, or menthone. Although seasonal variation influenced metabolite accumulation, genotype was the primary determinant of phytochemical composition. Carvone‐rich *M. spicata s*pecies are well‐suited for the flavor and fragrance industries, whereas *M. × piperita* chemotypes rich in menthol or menthone are valuable for use in pharmaceutical, oral care, and confectionery products. The dominance of piperitenone oxide in MPPS reveals a less common but potentially exploitable chemotype for specialized applications. The strong antioxidant potential of peppermint species further supports their use as a natural source of bioactive compounds. The present study demonstrates the dual influence of genotype and growing season on *Mentha* phytochemistry, providing a foundation for the optimized utilization of mint cultivars in the food, pharmaceutical, and cosmetic industries.

## 4 Experimental Section

### 4.1 Plant Material

Five *Mentha* genotypes were analyzed in this study: MS2 ‐ *Mentha spicata* “2”, MSCU ‐ *Mentha spicata* “Corvinus University”, MPPP ‐ *Mentha × piperita* “Perpeta”, MPCU ‐ *Mentha × piperita* “Corvinus University”, and MPPS ‐ *Mentha × piperita* “Persephone”. Plants were cultivated at experimental field of Mendel University in Brno, Faculty of Horticulture in Lednice (48.7954925 N, 16.7987650 E, 173 m above sea level), Czech Republic. Species were authenticated on the basis of morphological features by Jarmila Neugebauerová according to literature [22, 23]. The plant material of *M. × piperita* was also authenticated according to the European part of the Czech Pharmacopoeia [24]. The plants were harvested in July during the growing seasons of 2022–2024. The aerial part of plants was harvested manually and dried under controlled conditions in a KONEL EST‐03S dryer (Czech Republic). The drying cycle was set to 48 h at a maximum temperature of 40°C and a final moisture content of 8%. Dried samples were stored in paper bags in a dark place until analysis. Prior to analysis, the samples were ground to a fine powder using IKA MF 10 basic at 4000 rpm, for a particle size of 2 mm.

### 4.2 Cultivation Conditions

Meteorological data for each growing season were recorded with local automatic sensors (Lednice, Czech Republic). During the growing season (from January to July), the average temperature was 11.4°C in 2022, 11.1°C in 2023, and 12.9°C in 2024, with the mean precipitation of 29.5, 40.4, and 48.3 mm, respectively. The complete meteorological dataset for each growing season is provided in Figure S1. *Mentha* was grown on a precisely defined square area (1.2 × 1.2 m^2^). The crop was dense, weeding was done by hand, and the crop was not irrigated. The experimental plot was characterized by neutral to slightly alkaline soil (pH 7.0–7.6). Soil nutrient levels were determined as 96–166 mg/kg for phosphorus, 323–706 mg/kg for potassium, and 329–509 mg/kg for magnesium Table S1 by the accredited laboratory (LITOLAB Ltd., Litovel, Czech Republic). Soil available nutrients (P, K, Ca, Mg, Na, and selected micronutrients) were determined using the Mehlich 3 extraction method [25].

### 4.3 Sample Preparation

For the determination of TPC, TFC, AA, and individual phenolic compounds by HPLC–DAD, methanolic extracts of samples were prepared. A portion (1 g) of each dried plant material was mixed with 25 mL of 75% methanol (v/v) using a stick blender and extracted at room temperature for 24 h. The obtained extracts were filtered, filled with methanol to a volume of 30 mL, and kept in the fridge until further analysis.

The EOs for GC–MS analysis were obtained by Clevenger‐type hydrodistillation, in accordance with the method described in the European part of the Czech Pharmacopoeia [24]. Each sample (30 g) of plant material was placed in a 1 L round‐bottom flask with 400 mL of distilled water. Distillation was performed at a constant boiling rate for 2 h, after which the volume of EO was recorded. The yield of EO was calculated as the volume of oil obtained by hydrodistillation and expressed as a percentage (%, v/w), relative to the weight of the dry plant sample. All extractions and distillations were performed in triplicate for each plant material.

### 4.4 UV/Vis Measurements

All experiments were performed using UV/Vis spectrophotometer, Cecil CE 9500 Super Aquarius (Cecil Instruments Ltd, Cambridge, UK), in the same quartz cells (1 cm path length) against a blank. The experiments were performed three times, and results were expressed as the mean ± standard deviation (SD).

TPC of individual extracts was determined applying the Folin–Ciocalteu test [26, 27] with some modifications [28]. Standard solutions of gallic acid were used for calibration curve construction, and the results were expressed as mg of gallic acid equivalent per gram of dry weight (mg GAE/g DW).

TFC of individual extracts was evaluated following the modified aluminum chloride colorimetric method using catechin as reference compound [28, 29]. The results were calculated as mg of catechin equivalent per g of dry weight (mg CE/g DW).

The AA was determined on the basis of the ability of antioxidants present in the samples to scavenge ^•^DPPH radical, according to the standard method described by Brand‐Williams et al. [30] with modifications as described by Kalisz et al. [28]. Trolox was employed as the reference standard, and radical scavenging activity of the samples was expressed as mg of Trolox equivalent per g of dry weight (mg TE/g DW).

### 4.5 Determination of Individual Phenolic Compounds by HPLC–DAD

The extracts were analyzed using an Agilent 1200 series HPLC–DAD system (Agilent Technologies, Santa Clara, CA, USA). Chromatographic separation was carried out on a Zorbax SB‐C18 column (4.6* × *150 mm^2^, 5 µm particle size; Agilent Technologies, Santa Clara, CA, USA), maintained at a constant temperature of 30°C. The mobile phase consisted of acetonitrile (Solvent A) and 0.1% formic acid in water (Solvent B), with the following gradient program: 5% A at 0 min; 60% A at 15 min; 100% A at 20 min; held at 100% A from 20 to 30 min. Prior to each run, the column was equilibrated with 5% A for 10 min. The flow rate was set at 0.7 mL/min, and the injection volume was 5 µL. Detection was performed at 260, 280, and 360 nm. Target compounds were identified by comparing their retention times with those of authentic standards and the standard addition method. Calibration solutions at five concentration levels (2.5–500 µg/mL) were prepared by diluting a stock solution of individual phenolic standards (1 mg/mL) in accordance with their expected concentrations in the extracts. Each calibration level was injected into the HPLC–DAD system in triplicate. Calibration curves were constructed using linear regression of the peak area against the concentration for each compound. All analytes showed excellent linearity, with correlation coefficients (*r* ^2^) exceeding 0.997 across the tested range. Limits of detection (LOD, S/N = 3) and limits of quantification (LOQ, S/N = 10) ranged from 0.040 to 0.100 and 0.133 to 0.333 µg/mL, respectively. Method repeatability, expressed as relative standard deviation (RSD, %), was below 1% for all compounds analyzed.

### 4.6 GC–MS Analysis of EOs

The GC–MS analyses of EOs were carried out using a Thermo Trace GC Ultra equipped with a TriPlus autosampler, coupled with an ion‐trap Polaris Q mass spectrometer (EI mode at 70 eV; Thermo Fisher Scientific Inc., Waltham, USA). The chromatographic separation was performed on the DB‐5 capillary column (30 m* × *0.25 mm i.d., film thickness 0.25 µm; Agilent J&W Scientific, Folsom, USA); the following temperature program was used: 45°C held for 2 min, then increased to 250°C at a rate of 10°C/min, then increased to 300°C at a rate of 30°C/min, and finally held at 300°C for 2 min. The carrier gas was helium with a flow rate of 0.8 mL/min. The sample (1 µL) dissolved in *n*‐hexane was injected according to a splitless mode. The mass scan range (*m*/*z*) was 50–650 amu, data acquired at full scan mode with solvent delay for 4.7 min. Both the injector and the transfer line temperatures were set at 225°C and ion source at 220°C. The data were analyzed using Xcalibur 2.2 (Thermo Fisher Scientific Inc., Waltham, MA, USA), with identification of the individual components performed by comparison with co‐injected pure standards and by matching the MS fragmentation patterns and retention indices with the built‐in libraries or literature data (Adams 2007) or commercial mass spectral libraries (NIST MS Search 2.0 library; National Institute of Standards and Technology, Gaithersburg, USA). The relative amounts (%) of compounds were calculated on the basis of the GC peak area.

### 4.7 Statistical Analysis

Microsoft Office Excel 2019 (Redmond, WA, USA) and software STATISTICA.CZ version 14 (StatSoft, Prague, Czech Republic) were used for statistical analysis. The multivariate analysis of *Mentha* samples was carried out subjecting all obtained data to PCA. All PCA analyses were performed using Jamovi (version 2.6), an open‐source statistical software (The jamovi project, 2025, https://www.jamovi.org).

## Author Contributions

**Helena Pluháčková**: conceptualization, methodology, supervision, data curation, visualization, writing – original draft preparation, and writing – review and editing. **Barbora Kudláčková**: conceptualization, methodology, supervision, formal analysis and investigation, data curation, visualization, writing – original draft preparation, and writing – review and editing. **Marián Šinko**: conceptualization, field experiments, writing – review and editing. **Markéta Michutová**: conceptualization, writing – review and editing. **Jarmila Neugebauerová**: conceptualization, field experiments, writing – review and editing. **Lenka Svojanovská**: conceptualization, methodology, supervision, formal analysis and investigation, data curation, visualization, writing – original draft preparation, and writing – review and editing.

## Conflicts of Interest

The authors declare no conflicts of interest.

## Supporting information




**Supporting File 1**: cbdv70971‐sup‐0001‐SuppMat.docx

## Data Availability

The data that support the findings of this study are available in the Supporting Information of this article.
