# Supplementary material for: Phytochemical Variability of Mentha L. Species Over Three Growing Seasons
Source: Chem Biodivers. 2026 Mar 2;23(3):e03674. doi: 10.1002/cbdv.202503674 (PMC12953208; doi:10.1002/cbdv.202503674)
Supplement: Supplementary file 1 — Supporting File 1: cbdv70971‐sup‐0001‐SuppMat.docx [file CBDV-23-e03674-s001.docx]

**Supplementary information**

Phytochemical Variability of *Mentha* L. Species Over Three Growing Seasons.

Helena Pluháčková,^a^ Barbora Kudláčková,^b^ Marián Šinko,^c^ Markéta Michutová,^a^ Jarmila Neugebauerová,^d^ and Lenka Svojanovská*^,b^

^a^ Department of Crop Science, Breeding and Plant Medicine, Mendel University in Brno, Zemědělská 1665/1, 613 00 Brno, Czech Republic

^b^ Institute of Analytical Chemistry of the Czech Academy of Sciences, Veveří 967/97, 602 00 Brno, Czech Republic, email: svojanovska@iach.cz

^c^ Landscape Research Institute, Květnové náměstí 391, 252 43 Průhonice, Czech Republic

^d^ Department of Vegetable Growing and Floriculture, Mendel University in Brno, Valtická 337, Lednice 691 44, Czech Republic

**Figure S1**: Monthly average temperature and precipitation in 2022–2024

**Table S1**. The soil characteristics in experimental years 2022–2024.

| **Analysis / Year** | **2022** | **2023** | **2024** |
| --- | --- | --- | --- |
| K (mg/kg) | 323 (high) | 706 (very high) | 407 (very high) |
| Ca (mg/kg) | 3216 (good–very high) | 5021 (very high) | 4720 (very high) |
| Mg (mg/kg) | 329 (very high) | 509 (very high) | 430 (very high) |
| P (mg/kg) | 96 (good) | 166 (high) | 117 (good–high) |
| KVK (mmol/kg) | – | 320 (very high) | 281 (high) |
| pH | 7.6 (alkaline) | 7.45 (alkaline) | 7.02 (neutral) |
| NH₄⁺-N (mg/kg DM) | – | 0.912 (–) | 2.95 (–) |
| NO₃⁻-N (mg/kg DM) | – | 2.98 (–) | 1.70 (–) |
| Mineral N (mg/kg DM) | – | 3.89 (–) | 4.65 (–) |

**Table S2**. Results of UV/Vis measurements (TPC, TFC, AA) for all *Mentha* samples during 2022, 2023 and 2024.

| **YEARS** |  | **MS2** | **MSCU** | **MPPP** | **MPCU** | **MPPS** |
| --- | --- | --- | --- | --- | --- | --- |
|  | TPC (mg GAE/g DW) | 30.47 ± 0.96 | 36.98 ± 2.07 | 41.38 ± 1.63 | 42.43 ± 2.15 | - |
| **2022** | TFC (mg CE/g DW) | 25.44 ± 2.24 | 32.74 ± 2.52 | 30.12 ± 2.42 | 42.44 ± 2.30 | - |
|  | AA (mg TE/g DW) | 40.46 ± 2.30 | 46.86 ± 2.23 | 46.48 ± 2.47 | 63.90 ± 2.87 | - |
|  | TPC (mg GAE/g DW) | 31.57 ± 0.60 | 28.92 ± 2.42 | 33.61 ± 0.78 | 37.53 ± 1.03 | 42.12 ± 0.43 |
| **2023** | TFC (mg CE/g DW) | 26.53 ± 1.47 | 23.48 ± 2.50 | 27.56 ± 1.07 | 33.66 ± 1.12 | 43.01 ± 0.24 |
|  | AA (mg TE/g DW) | 45.78 ± 1.34 | 40.09 ± 2.51 | 48.30 ± 1.13 | 55.25 ± 2.44 | 68.36 ± 1.68 |
|  | TPC (mg GAE/g DW) | 39.57 ± 1.75 | 37.96 ± 2.10 | 39.76 ± 0.14 | 55.23 ± 1.65 | 47.74 ± 2.23 |
| **2024** | TFC (mg CE/g DW) | 35.11 ± 0.78 | 32.13 ± 2.83 | 33.25 ± 0.51 | 41.57 ± 1.68 | 39.15 ± 1.02 |
|  | AA (mg TE/g DW) | 93.59 ± 2.40 | 90.91 ± 2.30 | 89.78 ± 2.61 | 103.93 ± 1.33 | 100.31 ± 2.30 |

Values are mean ± SD (n = 3). TPC = total phenolic compound in mg Gallic Acid Equivalent/g of dry weight, TFC = total flavonoids content in mg Catechin equivalent/g of dry weight, AA = antioxidant activity in mg Trolox Equivalent/g of dry weight. MS2 **=** *Mentha spicata* ‘2’, MSCU = *Mentha spicata* ‘Corvinus University’, MPPP = *Mentha x piperita* ‘Perpeta’, MPCU = *Mentha x piperita* ‘Corvinus University’, MPPS = *Mentha x piperita* ‘Persephone’.

**Table S3.** Chemical composition of EOs (% ± SD) in all *Mentha* samples in **2022** determined by GC-MS.

| **Peak**  **No.** | **RI Calc** | **RI Adams** | **Compound** | **MS2**  **% ± SD** | **MSCU**  **% ± SD** | **MPPP**  **% ± SD** | **MPCU**  **% ± SD** |
| --- | --- | --- | --- | --- | --- | --- | --- |
| 1 | 937 | 932 | α-pinene | 0.04 ± 0.01 | 0.12 ± 0.02 | 0.11 ± 0.01 | 0.11 ± 0.01 |
| 2 | 976 | 969 | sabinene | 0.12 ± 0.03 | 0.18 ± 0.01 | 0.26 ± 0.05 | 0.13 ± 0.02 |
| 3 | 982 | 974 | β-pinene | 0.11 ± 0.01 | 0.16 ± 0.02 | 0.29 ± 0.06 | 0.16 ± 0.01 |
| 4 | 990 | 988 | myrcene | 0.04 ± 0.00 | 0.09 ± 0.00 | 0.07 ± 0.01 | 0.04 ± 0.00 |
| 5 | 997 | 988 | 3-octanol | 0.13 ± 0.02 | 0.14 ± 0.00 | 0.07 ± 0.01 | 0.07 ± 0.02 |
| 6 | 1020 | 1014 | α-terpinene | - | 0.05 ± 0.00 | 0.17 ± 0.02 | - |
| 7 | 1028 | 1020 | p-cymene | - | 0.02 ± 0.00 | 0.06 ± 0.02 | - |
| 8 | 1033 | 1024 | limonene | 0.83 ± 0.01 | 1.44 ± 0.03 | 0.62 ± 0.01 | 0.72 ± 0.01 |
| 9 | 1037 | 1026 | eucalyptol | 0.31 ± 0.02 | 0.37 ± 0.04 | 1.72 ± 0.03 | 0.04 ± 0.02 |
| 10 | 1061 | 1054 | γ-terpinene | 0.10 ± 0.01 | 0.12 ± 0.01 | 0.38 ± 0.06 | 0.04 ± 0.00 |
| 11 | 1075 | 1065 | cis-sabinene hydrate | 0.16 ± 0.02 | 0.15 ± 0.00 | 0.86 ± 0.05 | 0.04 ± 0.01 |
| 12 | 1100 | 1095 | linalool | 0.10 ± 0.01 | 0.25 ± 0.10 | - | - |
| 14 | 1156 | 1141 | camphor | - | 0.04 ± 0.00 | - | 0.11 ± 0.02 |
| 15 | 1163 | 1148 | menthone | 3.35 ± 0.09 | 0.10 ± 0.00 | 53.76 ± 1.98 | 23.82 ± 0.67 |
| 16 | 1171 | 1159 | menthofuran | 0.03 ± 0.01 | 0.01 ± 0.00 | 1.21 ± 0.25 | - |
| 17 | 1172 | 1158 | iso-menthone | 0.34 ± 0.01 | 0.03 ± 0.01 | 3.94 ± 0.06 | 7.34 ± 0.36 |
| 18 | 1176 | 1161 | neo-menthol | 0.79 ± 0.04 | - | 19.41 ± 0.05 | 0.93 ± 0.02 |
| 19 | 1183 | 1167 | DL-menthol | - | - | 0.29 ± 0.04 | 56.21 ± 0.75 |
| 20 | 1187 | 1174 | terpinen-4-ol | 0.19 ± 0.01 | 0.13 ± 0.01 | 0.51 ± 0.06 | - |
| 21 | 1195 | 1184 | neoiso-menthol | - | - | 0.93 ± 0.06 | 0.10 ± 0.00 |
| 22 | 1200 | 1186 | α-terpineol | 0.07 ± 0.00 | 0.11 ± 0.03 | 0.06 ± 0.01 | 0.20 ± 0.01 |
| 23 | 1204 | 1191 | cis-dihydrocarvone | 12.17 ± 1.45 | 12.08 ± 0.21 | 0.53 ± 0.07 | - |
| 24 | 1211 | 1200 | trans-dihydrocarvone | 0.32 ± 0.00 | 0.35 ± 0.02 | - | - |
| 25 | 1224 | 1215 | trans-carveol | 0.12 ± 0.01 | 0.12 ± 0.01 | - | - |
| 26 | 1238 | 1226 | cis-carveol | 0.21 ± 0.02 | 0.21 ± 0.01 | - | - |
| 27 | 1246 | 1233 | pulegone | 0.08 ± 0.01 | - | 2.09 ± 0.31 | - |
| 28 | 1252 | 1239 | carvone | 68.00 ± 0.70 | 71.27 ± 0.68 | 2.15 ± 0.47 | 0.06 ± 0.02 |
| 29 | 1262 | 1249 | piperitone | 0.09 ± 0.01 | - | 2.17 ± 0.23 | 6.13 ± 0.05 |
| 30 | 1270 | 1259 | cis-carvone oxide | 0.19 ± 0.03 | 0.18 ± 0.01 | - | - |
| 31 | 1276 | 1271 | neo-menthyl acetate | 0.08 ± 0.01 | - | 1.79 ± 0.10 | - |
| 32 | 1282 | 1273 | trans-carvone oxide | 0.27 ± 0.05 | 0.25 ± 0.02 | - | - |
| 33 | 1291  +1293 | 1289  + 1294 | Thymol  + menthyl acetate | - | - | 0.96 ± 0.01 | 0.22 ± 0.01 |
| 34 | 1299 | 1284* | dihydroedulan II | 0.11 ± 0.01 | 0.16 ± 0.00 | 0.12 ± 0.01 | - |
| 35 | 1304 | 1292* | dihydroedulan I | 0.08 ± 0.01 | 0.11 ± 0.00 | 0.19 ± 0.02 | - |
| 36 | 1311 | 1304 | iso-methyl acetate | - | - | 0.25 ± 0.01 | - |
| 37 | 1328 | 1346 | terpinyl acetate | 0.86 ± 0.06 | 0.68 ± 0.01 | - | - |
| 38 | 1341 | 1335 | δ-elemene | 0.05 ± 0.01 | 0.05 ± 0.00 | - | 0.03 ± 0.01 |
| 39 | 1349 | 1340 | piperitenone | - | - | - | - |
| 40 | 1361 | 1365 | carvyl acetate | 0.08 ± 0.01 | 0.08 ± 0.00 | - | - |
| 41 | 1369 | 1366 | piperitenone oxide | - | - | - | - |
| 42 | 1386 | 1373 | α-ylangene | 0.04 ± 0.00 | 0.05 ± 0.01 | 0.03 ± 0.00 | 0.02 ± 0.00 |
| 43 | 1390 | 1390 | trans-jasmone | 0.08 ± 0.01 | 0.04 ± 0.01 | - | - |
| 44 | 1396  +1398 | 1387  + 1389 | β-bourbonene  + β-elemene | 1.00 ± 0.19 | 2.38 ± 0.02 | - | 1.12 ± 0.06 |
| 45 | 1399 | 1392 | cis-jasmone | 0.90 ± 0.07 | 0.99 ± 0.01 | - | - |
| 46 | 1405 | 1641* | cinerolone | - | - | - | - |
| 47 | 1419 | 1409 | α-gurjunene | 0.13 ± 0.01 | 0.14 ± 0.00 | - | - |
| 48 | 1431 | 1413 | β-funebrene | 0.13 ± 0.01 | 0.25 ± 0.03 | 0.13 ± 0.01 | 0.08 ± 0.02 |
| 49 | 1434 | 1417 | β-caryophyllene | 2.31 ± 0.01 | 2.47 ± 0.06 | 2.39 ± 0.28 | 1.32 ± 0.01 |
| 50 | 1442 | 1430 | β-copaene | 0.32 ± 0.00 | 0.31 ± 0.00 | 0.08 ± 0.00 | 0.06 ± 0.00 |
| 51 | 1455 | 1439 | aromadendrene | - | - | 0.10 ± 0.00 | - |
| 52 | 1457 | 1442 | 6,9-guaiadiene | 0.62 ± 0.01 | 0.60 ± 0.02 | 0.24 ± 0.01 | 0.08 ± 0.00 |
| 53 | 1470 | 1452 | α-humulene | 0.40 ± 0.00 | 0.42 ± 0.01 | 0.12 ± 0.01 | 0.06 ± 0.01 |
| 54 | 1475 | 1461 | cis-cadina-1(6),4-diene | 0.76 ± 0.05 | 0.79 ± 0.01 | 0.20 ± 0.01 | - |
| 55 | 1482 | 1471 | dauca-5,8-diene | 0.18 ± 0.00 | 0.19 ± 0.02 | - | 0.07 ± 0.07 |
| 56 | 1494 | 1480 | germacrene D | 1.52 ± 0.09 | 1.49 ± 0.14 | 2.93 ± 0.27 | 0.67 ± 0.01 |
| 57 | 1508 | 1500  + 1500 | α-muurolene  + bicyclogermacrene | 0.37 ± 0.01 | 0.41 ± 0.05 | - | 0.27 ± 0.03 |
| 58 | 1525 | 1513 | γ-cadinene | 0.19 ± 0.04 | 0.15 ± 0.01 | 0.05 ± 0.01 | 0.02 ± 0.02 |
| 59 | 1528 | 1522 | δ-cadinene | 0.10 ± 0.02 | 0.14 ± 0.02 | 0.11 ± 0.01 | 0.05 ± 0.00 |
| 60 | 1532 | 1528 | cis-calamenene | 0.88 ± 0.03 | 0.89 ± 0.01 | 0.13 ± 0.01 | - |
| 61 | 1548 | 1537 | α-cadinene | 0.17 ± 0.01 | 0.15 ± 0.00 | - | - |
| 62 | 1592 | 1577 | spathulenol | 0.16 ± 0.00 | 0.19 ± 0.00 | - | 0.04 ± 0.01 |
| 63 | 1599 | 1582 | caryophyllene oxide | 0.13 ± 0.03 | 0.11 ± 0.00 | - | 0.06 ± 0.01 |
| 64 | 1613 | 1592 | viridiflorol | 0.09 ± 0.01 | 0.09 ± 0.01 | 0.23 ± 0.02 | - |
| 65 | 1630 | 1618 | 1,10-di-epi-cubenol | 0.39 ± 0.04 | 0.35 ± 0.02 | 0.09 ± 0.00 | - |
| 66 | 1656 | 1644 | α-muurolol | 0.11 ± 0.01 | 0.10 ± 0.01 | - | - |
| 67 | 1669 | 1652 | α-cadinol | 0.21 ± 0.04 | 0.18 ± 0.01 | 0.07 ± 0.00 | - |

Peak No. = Peak number; RI calc = Retention index calculated with respect to homologous series of n-alkanes (C9-C23) on a DB-5 column; RI lit = Retention index from literature - entered from Robert P. Adam’s library or * from NIST. MS2 - *Mentha spicata* ‘2’; MSCU - *Mentha spicata* ‘Corvinus University’; MPPP - *Mentha x piperita* ‘Perpeta’; MPCU - *Mentha x piperita* ‘Corvinus University’. Results are reported in % ± SD (standard deviation) as the mean of three experiments.

**Table S4**. Chemical composition of EOs (% ± SD) in all *Mentha* samples in **2023** determined by GC-MS.

| **Peak**  **No.** | **RI Calc** | **RI Adams** | **Compound** | **MS2**  **% ± SD** | **MSCU**  **% ± SD** | **MPPP**  **% ± SD** | **MPCU**  **% ± SD** | **MPPS**  **% ± SD** |
| --- | --- | --- | --- | --- | --- | --- | --- | --- |
| 1 | 937 | 932 | α-pinene | 0.22 ± 0.01 | 0.24 ± 0.01 | 0.36 ± 0.03 | 0.17 ± 0.01 | 0.06 ± 0.00 |
| 2 | 976 | 969 | sabinene | 0.18 ± 0.02 | 0.14 ± 0.01 | 0.23 ± 0.01 | 0.08 ± 0.00 | 0.07 ± 0.00 |
| 3 | 982 | 974 | β-pinene | 0.23 ± 0.00 | 0.19 ± 0.02 | 1.14 ± 0.02 | 0.14 ± 0.01 | 0.10 ± 0.01 |
| 4 | 990 | 988 | myrcene | 0.17 ± 0.01 | 0.11 ± 0.04 | 0.22 ± 0.01 | 0.12 ± 0.01 | 0.62 ± 0.01 |
| 5 | 997 | 988 | 3-octanol | 0.17 ± 0.01 | 0.11 ± 0.02 | 0.03 ± 0.00 | 0.09 ± 0.00 | - |
| 6 | 1020 | 1014 | α-terpinene | - | - | 0.30 ± 0.02 | 0.05 ± 0.01 | 0.02 ± 0.00 |
| 7 | 1028 | 1020 | p-cymene | - | - | 0.18 ± 0.01 | 0.05 ± 0.01 | - |
| 8 | 1033 | 1024 | limonene | 4.87 ± 0.02 | 2.08 ± 0.22 | 0.96 ± 0.03 | 1.54 ± 0.07 | 0.34 ± 0.00 |
| 9 | 1037 | 1026 | eucalyptol** | 0.70 ± 0.05 | 0.44 ± 0.01 | 3.64 ± 0.02 | 0.05 ± 0.01 | 1.88 ± 0.02 |
| 10 | 1061 | 1054 | γ-terpinene | 0.14 ± 0.00 | 0.18 ± 0.02 | 0.50 ± 0.04 | 0.03 ± 0.00 | 0.07 ± 0.00 |
| 11 | 1075 | 1065 | cis-sabinene hydrate | 0.35 ± 0.06 | 0.21 ± 0.01 | 1.04 ± 0.14 | 0.03 ± 0.00 | 0.17 ± 0.00 |
| 12 | 1100 | 1095 | linalool | 0.11 ± 0.01 | 0.19 ± 0.01 | - | - | 0.06 ± 0.00 |
| 13 | 1130 | 1128 | allo-ocimene | - | - | - | - | 0.14 ± 0.01 |
| 14 | 1156 | 1141 | camphor | - | 0.12 ± 0.08 | - | 0.09 ± 0.01 | - |
| 15 | 1163 | 1148 | menthone | 0.51 ± 0.18 | 1.57 ± 0.39 | 63.57 ± 0.77 | 29.70 ± 0.42 | - |
| 16 | 1171 | 1159 | menthofuran | 0.03 ± 0.01 | 0.02 ± 0.01 | 1.26 ± 0.24 | - | - |
| 17 | 1172 | 1158 | iso-menthone | 0.13 ± 0.01 | 0.39 ± 0.12 | 5.70 ± 0.12 | 9.88 ± 0.07 | - |
| 18 | 1176 | 1161 | neo-menthol | 0.23 ± 0.05 | 0.25 ± 0.11 | 10.05 ± 0.20 | 0.65 ± 0.06 | - |
| 19 | 1183 | 1167 | DL-menthol | 0.24 ± 0.04 | 1.03 ± 0.31 | 0.20 ± 0.01 | 46.48 ± 1.60 | - |
| 20 | 1187 | 1174 | terpinen-4-ol | 0.19 ± 0.00 | 0.15 ± 0.05 | 0.55 ± 0.08 | - | 0.10 ± 0.01 |
| 21 | 1195 | 1184 | neoiso-menthol | - | - | 0.11 ± 0.01 | 0.08 ± 0.01 | - |
| 22 | 1200 | 1186 | α-terpineol | 0.06 ± 0.00 | 0.07 ± 0.02 | 0.10 ± 0.02 | 0.27 ± 0.05 | 0.11 ± 0.02 |
| 23 | 1204 | 1191 | cis-dihydrocarvone | 7.28 ± 0.02 | 6.60 ± 0.93 | - | - | 0.09 ± 0.02 |
| 24 | 1211 | 1200 | trans-dihydrocarvone | 0.18 ± 0.01 | 0.13 ± 0.02 | - | - | - |
| 25 | 1224 | 1215 | trans-carveol | 0.10 ± 0.01 | 0.12 ± 0.01 | - | - | - |
| 26 | 1238 | 1226 | cis-carveol | 0.18 ± 0.01 | 0.33 ± 0.00 | - | - | - |
| 27 | 1246 | 1233 | pulegone | - | - | 0.35 ± 0.01 | - | - |
| 28 | 1252 | 1239 | carvone | 71.37 ± 0.23 | 64.82 ± 3.30 | - | 0.68 ± 0.09 | 3.19 ± 0.89 |
| 29 | 1262 | 1249 | piperitone | 0.23 ± 0.04 | 0.19 ± 0.09 | 1.85 ± 0.01 | 6.44 ± 0.93 | 0.08 ± 0.01 |
| 30 | 1270 | 1259 | cis-carvone oxide | 0.23 ± 0.03 | 0.33 ± 0.06 | - | - | 0.10 ± 0.01 |
| 31 | 1276 | 1271 | neo-menthyl acetate | - | 0.05 ± 0.03 | 1.09 ± 0.10 | 0.08 ± 0.01 | - |
| 32 | 1282 | 1273 | trans-carvone oxide | 0.38 ± 0.01 | 0.61 ± 0.07 | - | - | - |
| 33 | 1291  + 1293 | 1289  + 1294 | thymol  + menthyl acetate | - | - | 0.71 ± 0.04 | 0.07 ± 0.01 | - |
| 34 | 1299 | 1284* | dihydroedulan II | 0.19 ± 0.01 | 0.26 ± 0.01 | 0.15 ± 0.02 | - | 0.13 ± 0.01 |
| 35 | 1304 | 1292* | dihydroedulan I | 0.16 ± 0.01 | 0.13 ± 0.00 | 0.14 ± 0.04 | - | 0.32 ± 0.02 |
| 36 | 1311 | 1304 | iso-methyl acetate | - | - | 0.10 ± 0.00 | - | - |
| 37 | 1328 | 1346 | terpinyl acetate | 0.14 ± 0.00 | 0.25 ± 0.07 | - | - | - |
| 38 | 1341 | 1335 | δ-elemene | 0.04 ± 0.00 | 0.12 ± 0.06 | - | 0.04 ± 0.00 | 0.06 ± 0.00 |
| 39 | 1349 | 1340 | piperitenone | - | - | - | - | - |
| 40 | 1361 | 1365 | carvyl acetate | 0.06 ± 0.00 | 0.08 ± 0.03 | - | - | - |
| 41 | 1369 | 1366 | piperitenone oxide | - | - | - | - | 62.93 ± 3.16 |
| 42 | 1386 | 1373 | α-ylangene | 0.04 ± 0.00 | - | 0.03 ± 0.00 | - | 0.16 ± 0.01 |
| 43 | 1390 | 1390 | trans-jasmone | 0.07 ± 0.00 | - | - | - | - |
| 44 | 1396  + 1398 | 1387  + 1389 | β-bourbonene  + β-elemene | 1.45 ± 0.04 | 1.37 ± 0.31 | 0.42 ± 0.05 | 1.13 ± 0.01 | 1.70 ± 0.01 |
| 45 | 1399 | 1392 | cis-jasmone | 0.49 ± 0.01 | 0.73 ± 0.01 | - | - | 0.41 ± 0.02 |
| 46 | 1405 | 1641* | cinerolone | - | - | - | - | 0.13 ± 0.01 |
| 47 | 1419 | 1409 | α-gurjunene | 0.14 ± 0.01 | 0.26 ± 0.04 | - | - | - |
| 48 | 1431 | 1413 | β-funebrene | 0.10 ± 0.00 | 0.16 ± 0.00 | 0.03 ± 0.01 | - | 0.10 ± 0.01 |
| 49 | 1434 | 1417 | β-caryophyllene | 2.16 ± 0.07 | 3.03 ± 0.56 | 1.49 ± 0.16 | 1.03 ± 0.07 | 4.51 ± 0.21 |
| 50 | 1442 | 1430 | β-copaene | 0.37 ± 0.01 | 0.62 ± 0.02 | 0.05 ± 0.00 | 0.07 ± 0.01 | 0.31 ± 0.02 |
| 51 | 1455 | 1439 | aromadendrene | - | - | 0.06 ± 0.00 | - | 0.09 ± 0.01 |
| 52 | 1457 | 1442 | 6,9-guaiadiene | 0.42 ± 0.04 | 0.56 ± 0.04 | 0.07 ± 0.01 | - | 0.96 ± 0.08 |
| 53 | 1470 | 1452 | α-humulene | 0.36 ± 0.02 | 0.89 ± 0.33 | 0.07 ± 0.01 | - | 0.28 ± 0.04 |
| 54 | 1475 | 1461 | cis-cadina-1(6),4-diene | 0.73 ± 0.06 | 0.99 ± 0.01 | 0.13 ± 0.02 | - | 1.70 ± 0.14 |
| 55 | 1482 | 1471 | dauca-5,8-diene | 0.18 ± 0.01 | 0.41 ± 0.09 | - | - | - |
| 56 | 1494 | 1480 | germacrene D | 1.84 ± 0.02 | 4.36 ± 1.34 | 2.01 ± 0.20 | 0.53 ± 0.04 | 14.80 ± 0.82 |
| 57 | 1508 | 1500  + 1500 | α-muurolene  + bicyclogermacrene | 0.38 ± 0.01 | 1.49 ± 0.63 | - | 0.28 ± 0.02 | 0.50 ± 0.02 |
| 58 | 1525 | 1513 | γ-cadinene | 0.15 ± 0.01 | 0.21 ± 0.02 | 0.06 ± 0.00 | 0.09 ± 0.01 | 0.28 ± 0.01 |
| 59 | 1528 | 1522 | δ-cadinene | 0.12 ± 0.00 | 0.30 ± 0.11 | 0.07 ± 0.00 | 0.09 ± 0.01 | 0.31 ± 0.01 |
| 60 | 1532 | 1528 | cis-calamenene | 0.74 ± 0.02 | 1.19 ± 0.09 | 0.05 ± 0.01 | - | 0.90 ± 0.10 |
| 61 | 1548 | 1537 | α-cadinene | 0.16 ± 0.01 | 0.30 ± 0.08 | 0.04 ± 0.00 | - | 0.32 ± 0.05 |
| 62 | 1592 | 1577 | spathulenol | 0.18 ± 0.01 | 0.55 ± 0.15 | - | 0.06 ± 0.01 | 0.23 ± 0.05 |
| 63 | 1599 | 1582 | caryophyllene oxide | 0.16 ± 0.01 | 0.28 ± 0.01 | - | 0.08 ± 0.01 | 0.14 ± 0.03 |
| 64 | 1613 | 1592 | viridiflorol | 0.10 ± 0.00 | - | 0.19 ± 0.02 | - | - |
| 65 | 1630 | 1618 | 1.10-di-epi-cubenol | 0.38 ± 0.01 | 0.76 ± 0.08 | 0.07 ± 0.02 | - | 0.65 ± 0.05 |
| 66 | 1656 | 1644 | α-muurolol | 0.08 ± 0.01 | - | - | - | 0.20 ± 0.02 |
| 67 | 1669 | 1652 | α-cadinol | 0.25 ± 0.01 | 0.49 ± 0.04 | 0.10 ± 0.01 | 0.08 ± 0.01 | 0.49 ± 0.05 |

Peak No. = Peak number; RI calc = Retention index calculated with respect to homologous series of n-alkanes (C9-C23) on a DB-5 column; RI lit = Retention index from literature - entered from Robert P. Adam’s library or * from NIST. MS2 - *Mentha spicata* ‘2’; MSCU - *Mentha spicata* ‘Corvinus University’; MPPP - *Mentha x piperita* ‘Perpeta’; MPCU - *Mentha x piperita* ‘Corvinus University’; MPPS - *Mentha x piperita* ‘Persephone’. ** in sample MPPS is it the sum of eucalyptol and ocimene (RI 1032). Results are reported in % ± SD (standard deviation) as the mean of three experiments.

**Table S5**. Chemical composition of EOs (% ± SD) in all *Mentha* samples in **2024** determined by GC-MS.

| **Peak**  **No.** | **RI Calc** | **RI Adams** | **Compound** | **MS2**  **% ± SD** | **MSCU**  **% ± SD** | **MPPP**  **% ± SD** | **MPCU**  **% ± SD** | **MPPS**  **% ± SD** |
| --- | --- | --- | --- | --- | --- | --- | --- | --- |
| 1 | 937 | 932 | α-pinene | 0.27 ± 0.02 | 0.36 ± 0.03 | 0.41 ± 0.03 | 0.30 ± 0.02 | 0.12 ± 0.02 |
| 2 | 976 | 969 | sabinene | 0.18 ± 0.01 | 0.24 ± 0.02 | 0.36 ± 0.02 | 0.25 ± 0.03 | 0.11 ± 0.02 |
| 3 | 982 | 974 | β-pinene | 0.40 ± 0.01 | 0.44 ± 0.04 | 0.96 ± 0.11 | 0.31 ± 0.03 | 0.21 ± 0.08 |
| 4 | 990 | 988 | myrcene | 0.19 ± 0.02 | 0.13 ± 0.02 | 0.10 ± 0.01 | - | 0.31 ± 0.04 |
| 5 | 997 | 988 | 3-octanol | 0.09 ± 0.01 | 0.15 ± 0.00 | - | - | - |
| 6 | 1020 | 1014 | α-terpinene | - | 0.06 ± 0.00 | 0.22 ± 0.02 | - | 0.03 ± 0.00 |
| 7 | 1028 | 1020 | p-cymene | - | 0.03 ± 0.00 | 0.35 ± 0.04 | - | - |
| 8 | 1033 | 1024 | limonene | 6.72 ± 0.08 | 7.05 ± 0.13 | 0.92 ± 0.03 | 1.25 ± 0.07 | 0.37 ± 0.15 |
| 9 | 1037 | 1026 | eucalyptol** | 1.14 ± 0.01 | 0.74 ± 0.09 | 3.50 ± 0.11 | 0.36 ± 0.03 | 1.30 ± 0.20 |
| 10 | 1061 | 1054 | γ-terpinene | 0.09 ± 0.01 | 0.10 ± 0.01 | 0.48 ± 0.03 | - | 0.09 ± 0.01 |
| 11 | 1075 | 1065 | cis-sabinene hydrate | 0.10 ± 0.02 | 0.13 ± 0.01 | 0.28 ± 0.05 | - | 0.02 ± 0.01 |
| 12 | 1100 | 1095 | linalool | 0.32 ± 0.03 | 0.09 ± 0.00 | - | - | 0.08 ± 0.00 |
| 13 | 1130 | 1128 | allo-ocimene | - | - | - | - | 0.07 ± 0.00 |
| 14 | 1156 | 1141 | camphor | - | - | - | 0.17 ± 0.01 | - |
| 15 | 1163 | 1148 | menthone | 0.27 ± 0.03 | 0.31 ± 0.03 | 57.21 ± 1.84 | 32.29 ± 0.59 | 1.81 ± 0.68 |
| 16 | 1171 | 1159 | menthofuran | 0.02 ± 0.01 | 0.03 ± 0.01 | 1.25 ± 0.15 | - | 0.49 ± 0.17 |
| 17 | 1172 | 1158 | iso-menthone | 0.03 ± 0.01 | 0.02 ± 0.01 | 4.52 ± 0.30 | 10.14 ± 0.00 | - |
| 18 | 1176 | 1161 | neo-menthol | 0.11 ± 0.01 | - | 8.72 ± 0.32 | 0.36 ± 0.04 | 0.40 ± 0.18 |
| 19 | 1183 | 1167 | DL-menthol | - | - | 0.19 ± 0.01 | 45.85 ± 2.55 | - |
| 20 | 1187 | 1174 | terpinen-4-ol | 0.14 ± 0.01 | 0.16 ± 0.01 | 1.02 ± 0.06 | - | 0.71 ± 0.25 |
| 21 | 1195 | 1184 | neoiso-menthol | - | - | 0.52 ± 0.04 | - | - |
| 22 | 1200 | 1186 | α-terpineol | 0.09 ± 0.01 | 0.08 ± 0.00 | 0.08 ± 0.01 | - | 0.34 ± 0.08 |
| 23 | 1204 | 1191 | cis-dihydrocarvone | 7.32 ± 0.07 | 10.20 ± 0.00 | 0.06 ± 0.01 | - | - |
| 24 | 1211 | 1200 | trans-dihydrocarvone | 0.16 ± 0.01 | 0.16 ± 0.02 | - | - | - |
| 25 | 1224 | 1215 | trans-carveol | 0.10 ± 0.01 | 0.29 ± 0.02 | - | - | - |
| 26 | 1238 | 1226 | cis-carveol | 0.19 ± 0.01 | 0.25 ± 0.02 | - | - | - |
| 27 | 1246 | 1233 | pulegone | - | - | 4.55 ± 0.23 | - | 0.21 ± 0.06 |
| 28 | 1252 | 1239 | carvone | 65.44 ± 1.78 | 60.13 ± 2.75 | 2.43 ± 0.14 | - | 0.20 ± 0.02 |
| 29 | 1262 | 1249 | piperitone | - | - | 3.12 ± 0.33 | 4.16 ± 0.64 | 0.22 ± 0.07 |
| 30 | 1270 | 1259 | cis-carvone oxide | 0.25 ± 0.02 | 0.30 ± 0.03 | - | - | 0.13 ± 0.02 |
| 31 | 1276 | 1271 | neo-menthyl acetate | - | - | 1.21 ± 0.09 | - | - |
| 32 | 1282 | 1273 | trans-carvone oxide | 0.40 ± 0.03 | 0.34 ± 0.03 | - | - | - |
| 33 | 1291  + 1293 | 1289  + 1294 | thymol  + menthyl acetate | - | - | 0.53 ± 0.06 | 0.15 ± 0.02 | - |
| 34 | 1299 | 1284* | dihydroedulan II | 0.09 ± 0.01 | 0.08 ± 0.00 | 0.10 ± 0.01 | - | 0.02 ± 0.00 |
| 35 | 1304 | 1292* | dihydroedulan I | 0.12 ± 0.01 | 0.10 ± 0.01 | 0.24 ± 0.01 | - | 0.34 ± 0.03 |
| 36 | 1311 | 1304 | iso-methyl acetate | - | - | 0.23 ± 0.01 | - | - |
| 37 | 1328 | 1346 | terpinyl acetate | 0.23 ± 0.02 | 0.34 ± 0.03 | - | - | - |
| 38 | 1341 | 1335 | δ-elemene | 0.05 ± 0.00 | 0.03 ± 0.00 | - | - | - |
| 39 | 1349 | 1340 | piperitenone | - | - | - | - | 0.19 ± 0.01 |
| 40 | 1361 | 1365 | carvyl acetate | 0.07 ± 0.00 | 0.06 ± 0.01 | - | - | - |
| 41 | 1369 | 1366 | piperitenone oxide | - | - | - | - | 70.48 ± 2.74 |
| 42 | 1386 | 1373 | α-ylangene | 0.25 ± 0.02 | 0.11 ± 0.01 | 0.08 ± 0.01 | - | 0.07 ± 0.01 |
| 43 | 1390 | 1390 | trans-jasmone | 0.13 ± 0.01 | - | - | - | - |
| 44 | 1396  + 1398 | 1387  + 1389 | β-bourbonene  + β-elemene | 1.74 ± 0.01 | 2.14 ± 0.22 | 1.05 ± 0.11 | 0.91 ± 0.03 | 0.61 ± 0.08 |
| 45 | 1399 | 1392 | cis-jasmone | 1.09 ± 0.01 | 0.91 ± 0.14 | - | - | 0.25 ± 0.04 |
| 46 | 1405 | 1641* | cinerolone | - | - | - | - | 0.27 ± 0.03 |
| 47 | 1419 | 1409 | α-gurjunene | 0.08 ± 0.01 | 0.09 ± 0.01 | - | - | - |
| 48 | 1431 | 1413 | β-funebrene | 0.21 ± 0.01 | 0.30 ± 0.02 | 0.16 ± 0.01 | - | - |
| 49 | 1434 | 1417 | β-caryophyllene | 1.76 ± 0.03 | 2.81 ± 0.16 | 3.31 ± 0.33 | 1.45 ± 0.03 | 5.22 ± 0.19 |
| 50 | 1442 | 1430 | β-copaene | 0.28 ± 0.02 | 0.34 ± 0.04 | 0.09 ± 0.01 | - | 0.17 ± 0.01 |
| 51 | 1455 | 1439 | aromadendrene | - | - | 0.08 ± 0.01 | - | 0.18 ± 0.01 |
| 52 | 1457 | 1442 | 6,9-guaiadiene | 0.43 ± 0.01 | 0.59 ± 0.07 | 0.14 ± 0.01 | 0.09 ± 0.01 | 0.13 ± 0.01 |
| 53 | 1470 | 1452 | α-humulene | 0.35 ± 0.04 | 0.41 ± 0.04 | 0.13 ± 0.01 | - | 0.22 ± 0.01 |
| 54 | 1475 | 1461 | cis-cadina-1(6),4-diene | 1.02 ± 0.02 | 1.35 ± 0.09 | 0.32 ± 0.04 | - | 1.43 ± 0.16 |
| 55 | 1482 | 1471 | dauca-5,8-diene | 0.12 ± 0.01 | 0.15 ± 0.03 | - | - | - |
| 56 | 1494 | 1480 | germacrene D | 2.33 ± 0.03 | 2.01 ± 0.14 | 4.80 ± 0.30 | 0.83 ± 0.05 | 5.68 ± 0.33 |
| 57 | 1508 | 1500  + 1500 | α-muurolene  + bicyclogermacrene | 0.63 ± 0.05 | 0.50 ± 0.02 | - | 0.35 ± 0.02 | 0.33 ± 0.03 |
| 58 | 1525 | 1513 | γ-cadinene | 0.17 ± 0.02 | 0.11 ± 0.02 | 0.11 ± 0.01 | - | 0.18 ± 0.02 |
| 59 | 1528 | 1522 | δ-cadinene | 0.14 ± 0.01 | 0.06 ± 0.00 | 0.16 ± 0.01 | 0.08 ± 0.01 | 0.09 ± 0.01 |
| 60 | 1532 | 1528 | cis-calamenene | 0.77 ± 0.04 | 0.94 ± 0.11 | 0.18 ± 0.02 | - | 0.93 ± 0.10 |
| 61 | 1548 | 1537 | α-cadinene | 0.20 ± 0.01 | 0.22 ± 0.01 | 0.08 ± 0.01 | - | 0.25 ± 0.01 |
| 62 | 1592 | 1577 | spathulenol | 0.32 ± 0.02 | 0.20 ± 0.01 | - | 0.06 ± 0.00 | 0.43 ± 0.01 |
| 63 | 1599 | 1582 | caryophyllene oxide | 0.18 ± 0.01 | 0.18 ± 0.02 | - | 0.07 ± 0.00 | 0.75 ± 0.02 |
| 64 | 1613 | 1592 | viridiflorol | 0.06 ± 0.00 | 0.06 ± 0.00 | 0.25 ± 0.03 | - | - |
| 65 | 1630 | 1618 | 1,10-di-epi-cubenol | 0.47 ± 0.02 | 0.63 ± 0.00 | 0.17 ± 0.02 | - | 1.01 ± 0.09 |
| 66 | 1656 | 1644 | α-muurolol | 0.10 ± 0.01 | 0.16 ± 0.01 | - | - | 0.51 ± 0.04 |
| 67 | 1669 | 1652 | α-cadinol | 0.29 ± 0.02 | 0.35 ± 0.01 | 0.16 ± 0.01 | 0.03 ± 0.00 | 0.83 ± 0.08 |

Peak No. = Peak number; RI calc = Retention index calculated with respect to homologous series of n-alkanes (C9-C23) on a DB-5 column; RI lit = Retention index from literature - entered from Robert P. Adam’s library or * from NIST. MS2 - *Mentha spicata* ‘2’; MSCU - *Mentha spicata* ‘Corvinus University’; MPPP - *Mentha x piperita* ‘Perpeta’; MPCU - *Mentha x piperita* ‘Corvinus University’; MPPS - *Mentha x piperita* ‘Persephone’. ** in sample MPPS is it the sum of eucalyptol and ocimene (RI 1032). Results are reported in % ± SD (standard deviation) as the mean of three experiments.


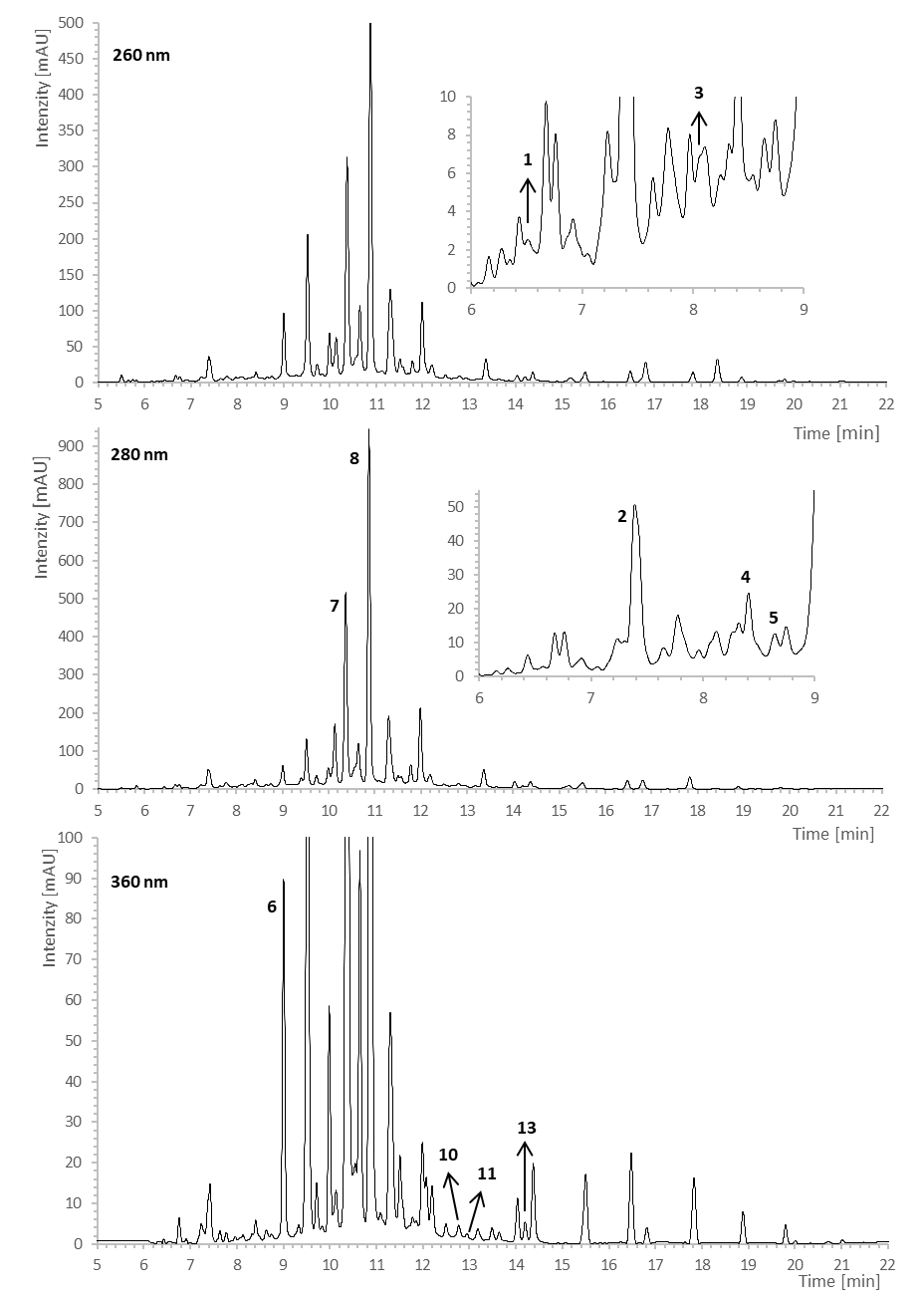


**Figure S2**: HPLC-DAD chromatogram of methanolic extract of *Mentha spicata* ‘2’ (2023). Peak identification: 1) protocatechuic acid; 2) chlorogenic acid; 3) p-hydroxybenzoic acid; 4) caffeic acid; 5) syringic acid; 6) rutin; 7) ferulic acid; 8) rosmarinic acid; 9) myricetin; 10) luteolin; 11) quercetin; 12) cinnamic acid; 13) apigenin.


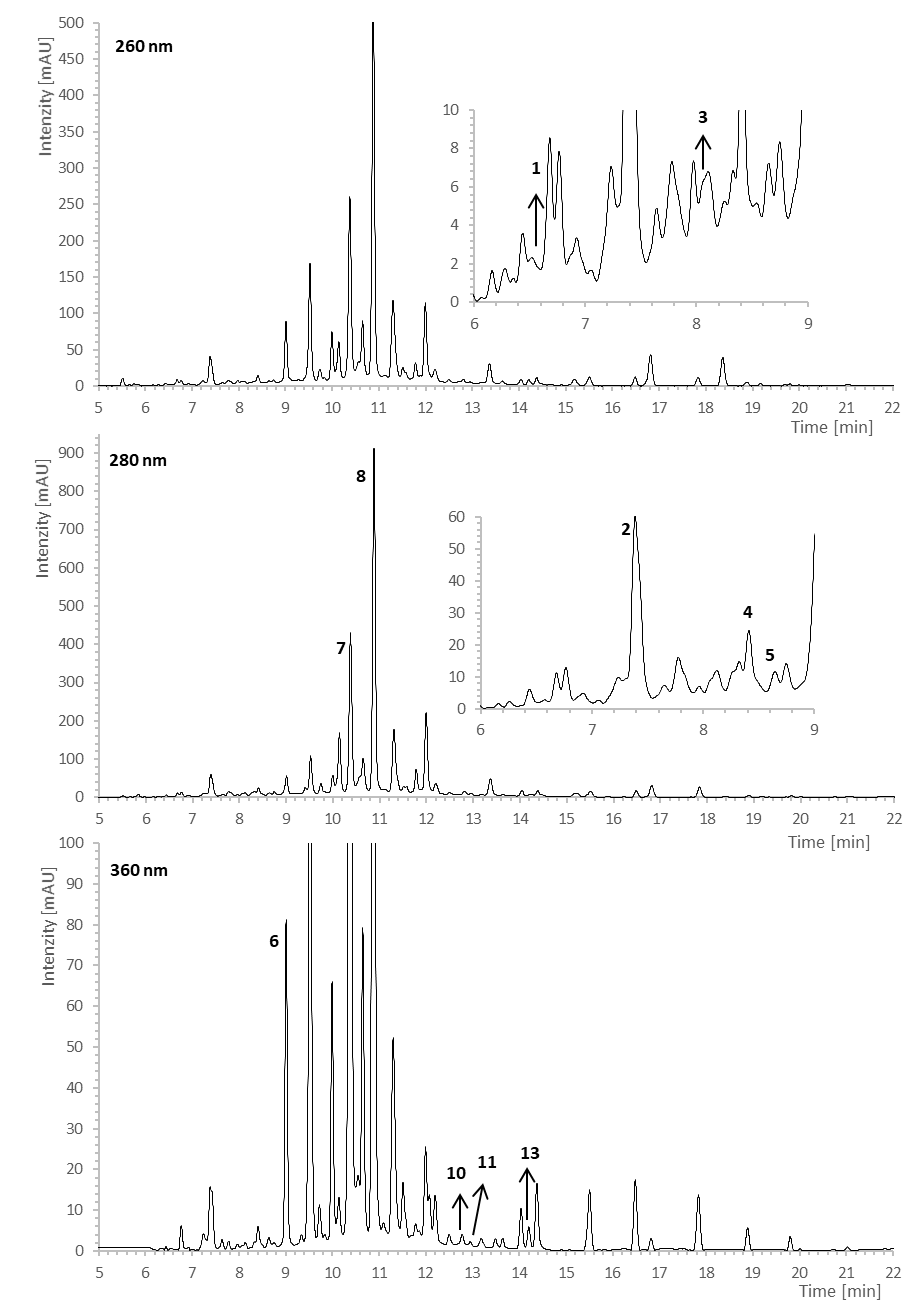


**Figure S3**: HPLC-DAD chromatogram of methanolic extract of *Mentha spicata* ‘Corvinus University’ (2023). Peak identification: 1) protocatechuic acid; 2) chlorogenic acid; 3) p-hydroxybenzoic acid; 4) caffeic acid; 5) syringic acid; 6) rutin; 7) ferulic acid; 8) rosmarinic acid; 9) myricetin; 10) luteolin; 11) quercetin; 12) cinnamic acid; 13) apigenin.


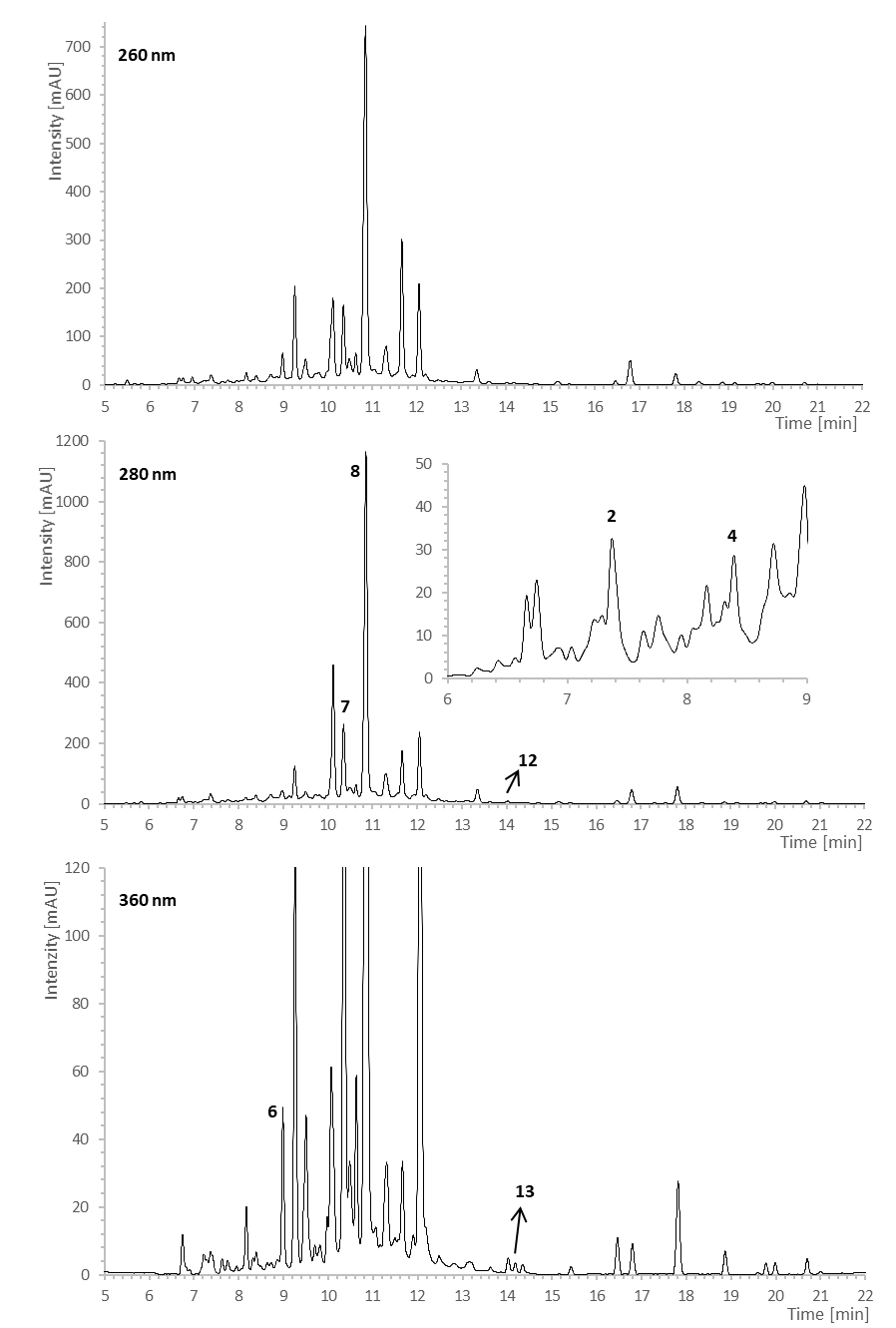


**Figure S4**: HPLC-DAD chromatogram of methanolic extract of *Mentha x piperita* ‘Perpeta’ (2023). Peak identification: 1) protocatechuic acid; 2) chlorogenic acid; 3) p-hydroxybenzoic acid; 4) caffeic acid; 5) syringic acid; 6) rutin; 7) ferulic acid; 8) rosmarinic acid; 9) myricetin; 10) luteolin; 11) quercetin; 12) cinnamic acid; 13) apigenin.


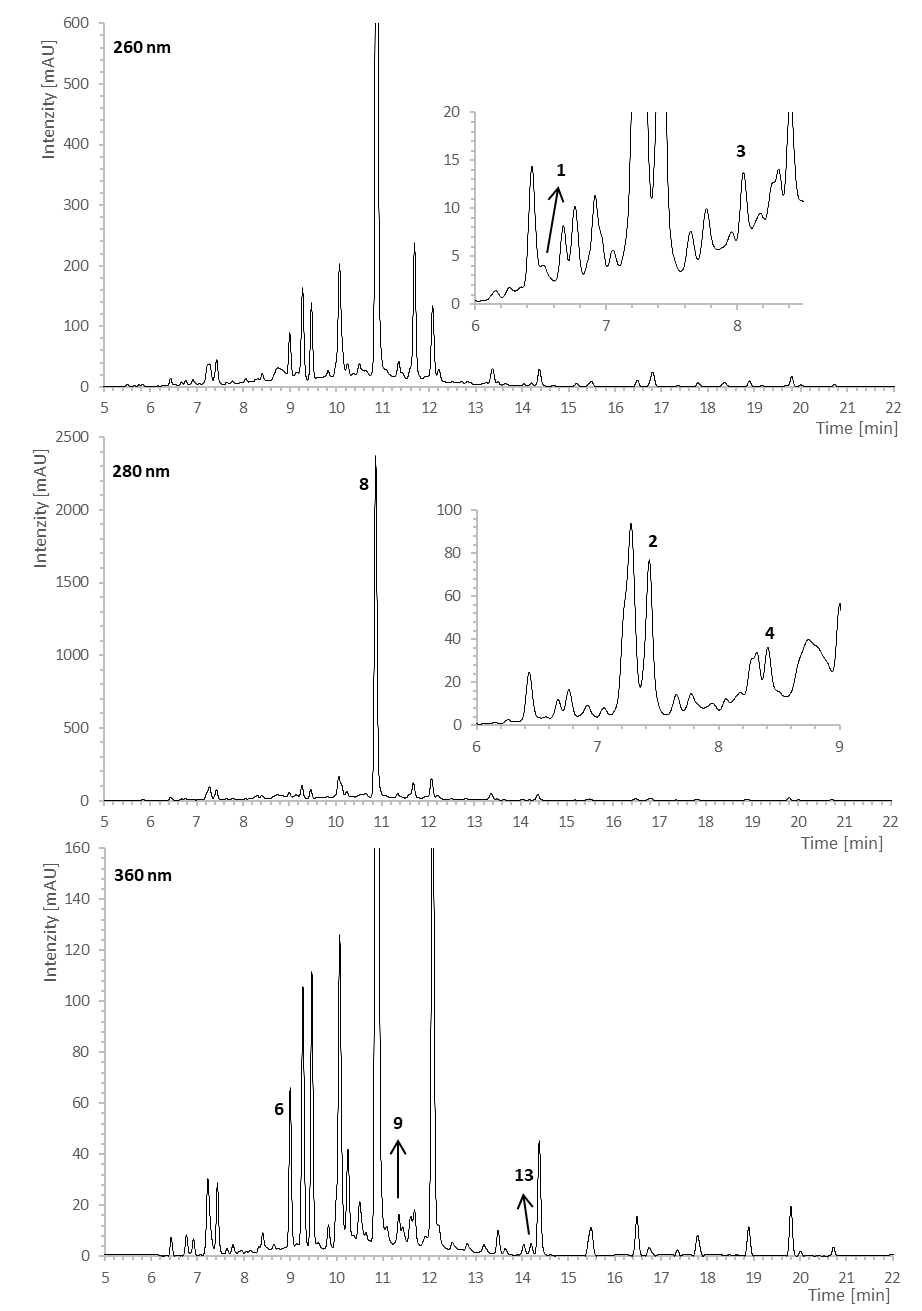


**Figure S5**: HPLC-DAD chromatogram of methanolic extract of *Mentha x piperita* ‘Corvinus University’ (2023). Peak identification: 1) protocatechuic acid; 2) chlorogenic acid; 3) p-hydroxybenzoic acid; 4) caffeic acid; 5) syringic acid; 6) rutin; 7) ferulic acid; 8) rosmarinic acid; 9) myricetin; 10) luteolin; 11) quercetin; 12) cinnamic acid; 13) apigenin.


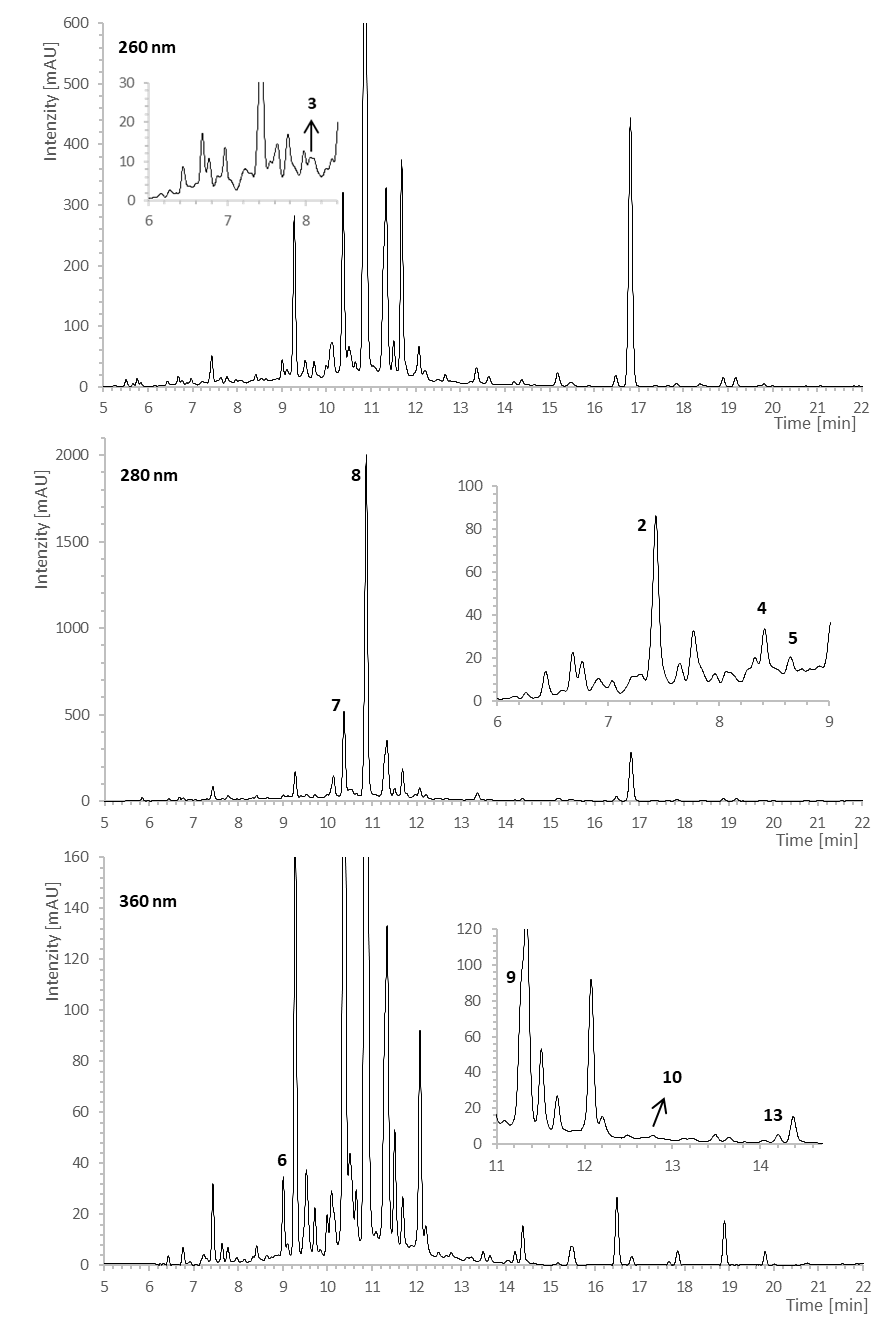


**Figure S6**: HPLC-DAD chromatogram of methanolic extract of *Mentha x piperita* ‘Persephone’ (2023). Peak identification: 1) protocatechuic acid; 2) chlorogenic acid; 3) p-hydroxybenzoic acid; 4) caffeic acid; 5) syringic acid; 6) rutin; 7) ferulic acid; 8) rosmarinic acid; 9) myricetin; 10) luteolin; 11) quercetin; 12) cinnamic acid; 13) apigenin.


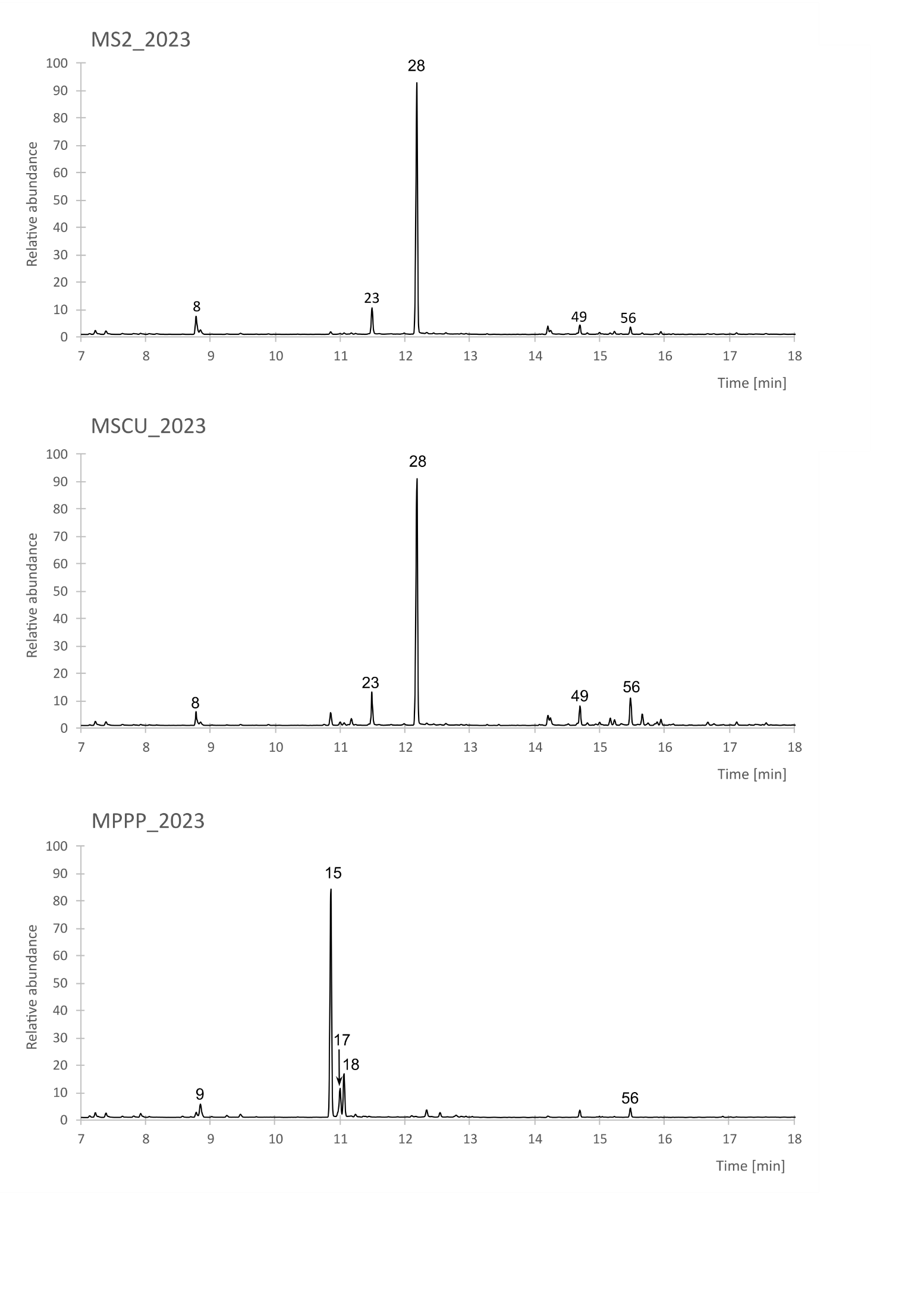


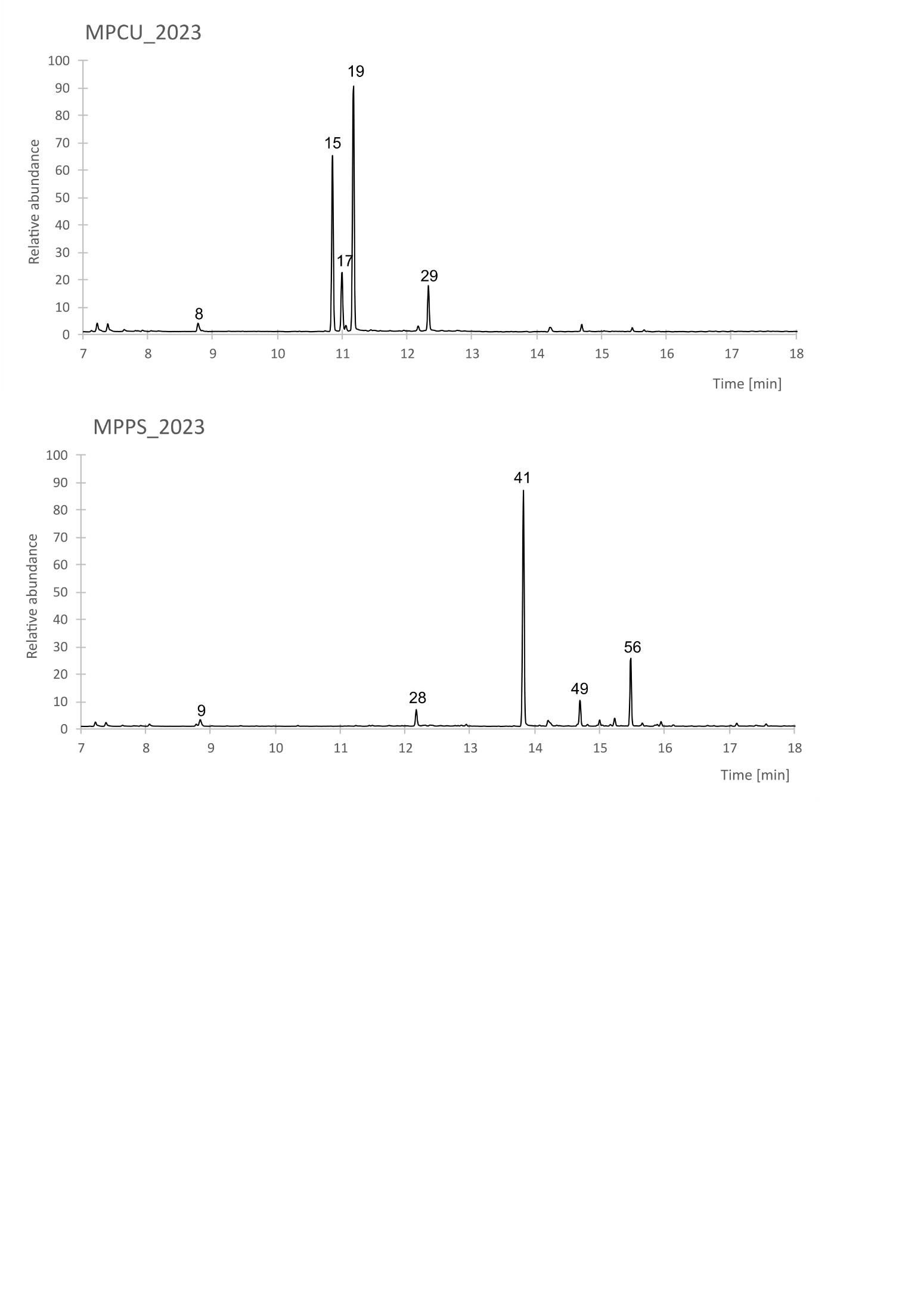


**Figure S7**: GC-MS chromatograms (TIC) of the EOs of *Mentha* samples in 2023. MS2_2023 = *Mentha spicata* ‘2’; MSCU_2023 = *Mentha spicata* ‘Corvinus University’; MPPP_2023 = *Mentha x piperita* ‘Perpeta’; MPCU_2023 = *Mentha x piperita* ‘Corvinus University’; MPPS_2023 = *Mentha x piperita* ‘Persephone’. The numbering corresponds to tables S2-S4. Peak identification of the five highest peaks: 8 - limonene, 9 - eucalyptol, 15 - menthone, 17 - iso-menthone, 18 - neo-menthol, 19 - DL-menthol, 23 - cis-dihydrocarvone, 28 - carvone, 29 - pirperitone, 41 - piperitenone oxide, 49 - β-caryophyllene, 56 - germacrene D.


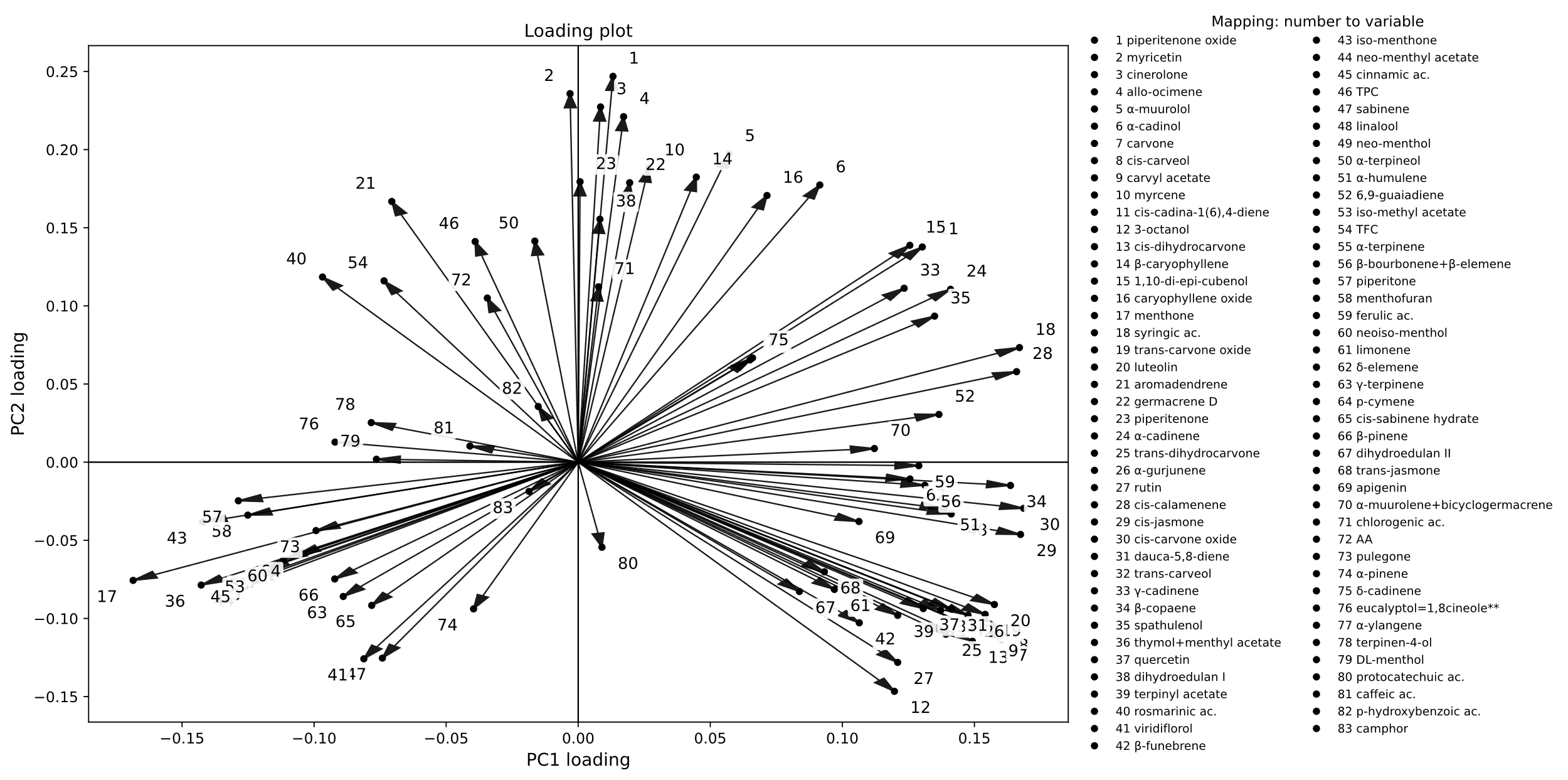


**Figure S8**: PCA of the chemical profiles of Mentha spicata and Mentha × piperita species across three years (2022–2024). Loading plot showing all variable loadings by absolute contribution to PC1/PC2.
